# Local‐ and landscape‐scale variables shape insect diversity in an urban biodiversity hot spot

**DOI:** 10.1002/eap.2089

**Published:** 2020-03-05

**Authors:** Benjamin J. Adams, Enjie Li, Christine A. Bahlai, Emily K. Meineke, Terrence P. McGlynn, Brian V. Brown

**Affiliations:** ^1^ Urban Nature Research Center Natural History Museum of Los Angeles County Los Angeles California 90007 USA; ^2^ Department of Biological Sciences Kent State University Kent Ohio 44242 USA; ^3^ Department of Entomology and Nematology University of California Davis California 95616 USA; ^4^ Department of Biology California State University Dominguez Hills Carson California 90747 USA; ^5^ Department of Entomology Natural History Museum of Los Angeles County Los Angeles California 90007 USA

**Keywords:** citizen science, community ecology, Diptera, Hymenoptera, Lepidoptera, Los Angeles

## Abstract

Local community structure is shaped by processes acting at local and landscape scales. The relative importance of drivers operating across different spatial scales is difficult to test without observations across regional or latitudinal gradients. Cities exhibit strong but predictable environmental gradients overlaying a mosaic of highly variable but repeated habitat types within a constrained area. Thus, cities present a unique opportunity to explore how both local and landscape factors influence local biotic communities. We used insect communities to examine the interactions among local environmental variables (such as temperature and relative humidity), local habitat characteristics (such as plant community composition), and broad‐scale patterns of urbanization (including biophysical, human‐built, and socioeconomic variables) on local insect abundance, species richness, and species composition in Los Angeles, a hot, dry, near‐desert city. After accounting for seasonal trends, insect species richness and abundance were highest in drier and hotter sites, but the magnitude of local environmental effects varied with the degree of urbanization. In contrast, insect species composition was best predicted by broad‐scale urbanization trends, with the more native communities occurring in less urbanized sites and more cosmopolitan insects occurring in highly urbanized sites. However, insect species richness and abundance were >30% higher and insect composition was similar across sites that hosted either native or drought‐tolerant plants, regardless of the degree of urbanization. These results demonstrate that urban insect biodiversity is a product of interacting mechanisms working at both local and landscape scales. However, local‐scale changes to urban habitats, such as cultivating plants that are adapted to the natural environment nearest the city, can positively impact urban biodiversity regardless of location.

## Introduction

Understanding the mechanisms that drive biodiversity across spatial and temporal scales is a fundamental goal of ecology (Levin 1992). Organisms interact with each other and experience habitat filters within localized areas that are embedded within broader environmental gradients (Kneitel and Chase 2004). Natural features like coastlines (Menge 1976) and mountains (Körner 2007) often are used to compare the importance of broad environmental gradients vs. local habitat variables on patterns of diversity within a constrained area that would normally only be possible by sampling over large, latitudinal gradients. Urban landscapes also present a unique, and relatively unexplored opportunity to test for broad and fine‐scale factors influencing local community structure, while presenting few of the logistical challenges associated with broad‐scale gradients (Pickett et al. 2017). In addition, patterns of biodiversity across cities are central to informing urban planning and restoration (McDonnell and MacGregor‐Fors 2016), endeavors that are of direct importance to human well‐being (Fuller et al. 2007) as more of the human population continues to move to cities (Seto et al. 2012).

Cities exhibit predictable, broad‐scale gradients overlaying a mosaic of variable but repeated habitats (Grimm et al. 2008). For example, moving from rural to more urbanized landscapes, average temperature typically increases due to urban heat islands effects (Oke 1973); nitrogen runoff often increases due to fertilization (Kaye et al. 2006); and hydrosystems can become less seasonally predictable due to modifications of waterways or water sources (Walsh et al. 2005). Within these broad urban gradients exists a fine‐scale mosaic of habitats (e.g., yards, parking lots, commercial districts, industrial warehouses) differentiated by variation in vegetation, resource availability, and ground and canopy cover (Pickett et al. 2017).

Urban biodiversity responds to both broad‐scale urban gradients and fine‐scale habitat heterogeneity independently. However, a knowledge gap exists with respect to how biodiversity responds to the interaction between these two scales (Goddard et al. 2010), which has only recently begun to receive more thorough attention (Kyrö et al. 2018, Anderson et al. 2019, Burdine and McCluney 2019). In general, increased urbanization corresponds with a decrease in species richness (Aronson et al. 2014) and the homogenization of plant and animal communities (Groffman et al. 2017). However, abundance of “urbanophile” species is often higher within cities (Shochat et al. 2010, Faeth et al. 2011) and cities can host rare or unknown species (Hartop et al. 2015, Longcore and Osborne 2015, Soanes and Lentini 2019), resulting in novel species assemblages (Sattler et al. 2011, Hall et al. 2017). Increased urbanization also can mitigate seasonal fluctuations in diversity normally associated with changes in precipitation or temperature (Bang and Faeth 2011, Andrade et al. 2017, Hung et al. 2017). Fine‐scale habitat heterogeneity can have drastic effects on plant phenology (McDonnell et al. 1997), animal physiology (Partecke et al. 2006, Tüzün et al. 2017), and trophic interactions (Faeth et al. 2005, Meineke et al. 2013, Dale and Frank 2018), which can ultimately affect local community assembly (Shochat et al. 2006, McGlynn et al. 2019). How local communities of organisms are affected by fine‐scale site characteristics across the broad‐scale urban gradient is often unaddressed because it requires replication of habitat types across the urban gradient (Goddard et al. 2010).

Insects are an excellent model for exploring patterns of diversity, especially within urban landscapes (McIntyre 2000, Bang and Faeth 2011). Insects are abundant, species‐rich, ecologically important, and display a wide variety of life‐history strategies (Triplehorn and Johnson 2005). Unlike urban plant communities (Faeth et al. 2011, Wheeler et al. 2017), insects also are not directly managed as part of the urbanization process except as pests (Rust and Su 2012). Thus, compared to plants, distributions of insects are driven more by the physical and resultant biotic environment than by planned human intervention (McIntyre et al. 2001). Additionally, short generation‐times and standardized collection and identification methods make insects tenable to ecological monitoring and highly responsive indicators of environmental heterogeneity and change across a landscape (McGeoch 1998, McIntyre 2000, McIntyre et al. 2001). Beyond their scientific and logistical benefits, insects also are economically important, providing billions of dollars annually in ecosystem services that include pollination, pest control, and decomposition (Losey and Vaughan 2006). Insects also serve as a powerful tool for facilitating community awareness of nature and science through citizen‐science projects and scientific education (Lucky et al. 2014, Walker et al. 2016), which are key to promoting conservation in urban ecosystems (Clark et al. 2016, Pickett et al. 2016).

Insect communities are influenced by broad‐scale impacts of urbanization and fine‐scale local processes (Niemelä et al. 2002, Bang and Faeth 2011, Egerer et al. 2017). At broad scales, insect species richness declines in more urbanized areas (McIntyre 2000). However, total insect abundance often increases in city greenspaces relative to natural landscapes due to higher abundance of generalist urbanophiles (including nonnative species) or the concentration of resources (Shochat et al. 2010, Faeth et al. 2011). Consistent water availability and more stable annual temperatures in urban landscapes can support higher abundances and decrease seasonal variation for insects like aphids and bees (Andrade et al. 2017, Hung et al. 2017). Urban warming also can drive higher herbivorous pest abundance via increased fecundity and reduced predation pressure (Dale and Frank 2014, Meineke et al. 2014). At the scale of a yard, park, or neighborhood, differences in local site characteristics and habitat quality have much more varied effects on insect communities. For example, high abundance of flowering plants (Bates et al. 2011) or a diverse plant community within a neighborhood or garden (Angold et al. 2006, Lerman et al. 2012, Hartop et al. 2018) increases the diversity of insect pollinators and ground beetles. However, cultivating native plant gardens at the scale of a yard does not always positively influence insect diversity when only locally adapted, nonnative plants are present (Matteson and Langellotto 2011; but see Burghardt et al. 2009, Narango et al. 2017). Insect responses to the interactions among site‐level characteristics and across an urban gradient are not often explored in combination but are likely important predictors of local community structure (Goddard et al. 2010 but see Bang and Faeth 2011).

Our primary objective was to explore how the interactions between broad‐scale urbanization and fine‐scale site differences shape patterns of insect diversity across Los Angeles County. Specifically, we examined how insect species richness, abundance, and species composition were affected by site‐specific environmental variables and local habitat characteristics placed within the context of a broad‐scale urbanization gradient. We also considered seasonal changes in insect communities because urbanization can minimize seasonal differences (Hung et al. 2017). We predicted that more urbanized sites would have lower overall species richness but support more cosmopolitan assemblages of insect urbanophiles (Faeth et al. 2011). At the fine scale, we expected consistently warmer and wetter sites (higher local temperatures and percent relative humidity) to host more diverse insect communities, especially within the most urbanized sites (Bang and Faeth 2011, Andrade et al. 2017). We predicted increased insect species richness and proportionally more native species at sites harboring native plants due to high endemism in California (Harrison 2013). We employed a contributory citizen‐science project model (Bonney et al. 2009) approach to our insect collections methods to facilitate community engagement and participation in this project (Brown et al. 2014).

## Methods

### Study sites

Los Angeles County (34.05° N, 118.24° W) is on the southwestern coast of the United States in the California Floristic Province, a global biodiversity hot spot (Cincotta et al. 2000). The area is characterized by its relatively warm, dry, Mediterranean‐like climate with high rates of endemism (Harrison 2013, Western Regional Climate Center 2018; Appendix S2: Fig. S1). The county is also home to more than 10 million people and is one of the largest urban centers in the United States (U.S. Census Bureau 2018). As part of an ongoing study of urban biodiversity in Los Angeles County (Brown et al. 2014), we collaborated with local residents, schools, and public institutions who provided yards and green spaces (as well as volunteers to change trap jars) at 30 sites across a large‐scale urban gradient for 1 yr (Appendix S2: Fig. S2). Sites typically consisted of urban and suburban backyards maintained under a variety of different landscaping regimes ranging from typical turf grass lawns with ornamental plants to xeriscaped yards with native grasses and shrubs.

To characterize broad‐scale urbanization patterns across Los Angeles county, we used the urban habitat classification scheme established by Li and colleagues (Li et al. 2019; Appendix S1). This classification scheme established nine distinct urban habitat types derived from 18 continuous environmental variables (including measurements of the biophysical landscapes, the human‐built environments, and socio‐economic structures). Urban types 1–4 represent less urbanized locations including natural areas, urban parks, and open green spaces. Types 5–9 represent more developed areas in the county but are differentiated by biophysical and anthropogenic characters such as annual rain fall and temperature patterns, population density, traffic density, and percent cover by impervious surfaces (Appendix S2: Table S1). Sites in this study are located in seven of the nine urban types (all except types 2 [wetlands habitats] and 7 [basin less developed areas]).

We used weather stations (HOBO U30 USB Weather Station Data Logger with S‐TMB‐M006 and S‐LIB‐M003 probes; Onset Computer Corporation, Bourne, Massachusetts, USA) to record fine‐scale environmental variables including air temperature, percent relatively humidity (RH), and solar radiation every five minutes for the entire year at each site. We used the weather station measurements to calculate the average daily mean, minimum, and maximum air temperatures and RH; and the average daily photoperiod for each site during collection periods. Due to intermittent temporary mechanical failures of the weather stations, local environmental data was occasionally interpolated from within a collection period by estimating the value of a missing parameter using its relationship to the same parameter at a site with similar microclimate (McGlynn et al. 2019). We down‐sampled high‐resolution weather station data to a weekly temporal resolution appropriate to our insect sampling regime.

We also characterized the habitat at each site using six ecologically relevant binary site descriptors that were also of interest to local residents. Specifically, we initially asked site hosts to characterize their yards based on the presence or absence of a turf grass lawn, native plants, drought‐tolerant plants, compost, mulched landscaping, and frequent watering (> once per week). We confirmed these binary characterizations via visual inspection of each yard in person during the course of the collection period.

### Insect collections

Local residents, students, and researchers collected insects using a Malaise trap (Townes 1972) placed at each site. Malaise traps passively collect insects in a systematic manner providing a standard sampling unit, and citizen‐science collaborators were responsible for changing the trap collection bottles each week. Traps were run continuously for all of 2014; however, each collection in this study represents only the insects accumulated over 7 ± 2 d (mean ± SD) at the beginning of each month at each of the 30 sites (*n* = 360 total samples). All insects were initially collected and stored in 95% ethanol. Based on their value as potential biological indicators of urbanization, varied life‐history traits, and the taxonomic expertise of the authors, we identified insects from three orders of interest: Diptera, Hymenoptera, and Lepidoptera. Within Diptera, we targeted five specific families of flies: Drosophilidae, Phoridae, Scatopsidae, Syrphidae, and Tipulidae. Within Hymenoptera, we focused exclusively on bees. Within Lepidoptera, we identified macrolepidotera including all butterflies and large moths. All individual insects from these groups were identified to species, species complex, or morphospecies. We recorded total abundance of individuals within a species (or minimum‐ranked taxa) and total species richness (treating each minimum‐ranked taxa as a species) within each collection. Voucher specimens are stored at the Natural History Museum of Los Angeles County, and in the case of phorid flies, the Cambridge University Museum of Zoology, the Smithsonian National Museum of Natural History, and the Museum of Comparative Zoology Collection.

### Species richness and abundance

We first created sample‐based species accumulation curves (SAC) for each of the seven focal taxa of flies, bees, and moths to evaluate our coverage of the insect communities. We visually assessed if SACs reached an asymptote and compared the number of species actually collected to the predicted maxima (*S*_est_, Chao1, 9,999 permutations) to determine if the sampling effort was sufficient (Colwell 2009). We also explored potential spatial autocorrelation in insect species occurrence by calculating Moran's *I* value for each month of collections based on insect species richness with an inverse distance matrix of site coordinates as the weighting factor (Paradis et al. 2004).

We used repeated measures linear models (Pinheiro et al. 2014) to assess the impact of broad‐scale urbanization (the urban typologies), the local environmental measurements (weather station data) and local ecological characteristics (binary site descriptors) on both insect species richness and insect abundance throughout the year. As the environmental variables recorded by the weather stations strongly correlated with each other, we input standardized measurements of all the environmental variables into Principal Component Analyses (PCA) for each season and used the scores from the first two PCA axes as proxies for the local environment. Urban type, PCA axes scores, and their interaction terms plus the six binary descriptors served as fixed effects in the models. Individual sites functioned as the repeated‐measure grouping factor across months (i.e., random effects).

**Figure 1:**
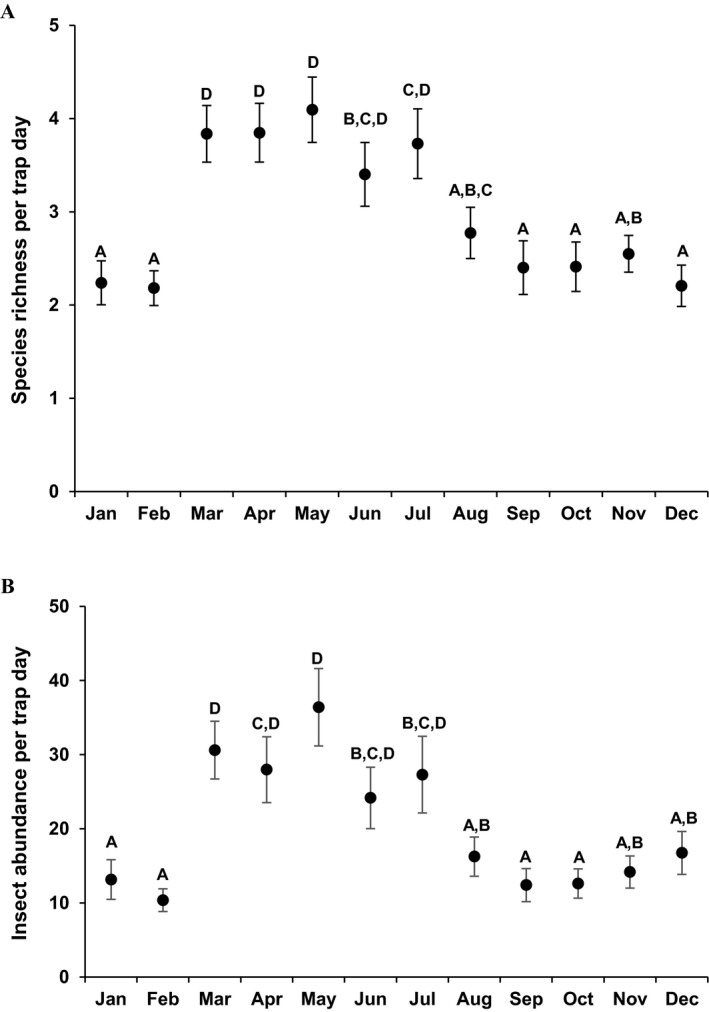
Raw (A) insect species richness and (B) insect abundance per trap day across all 12 months of the study. Values are mean ±SE. Values are mean ± SE. Different letters indicate significant difference in means (P≤0.05).

These initial tests using the whole year of data revealed clear seasonal trends in the local environmental parameters (e.g., expected seasonal differences in temperature, relative humidity, and solar period) and species richness (e.g., annual peaks in insect diversity), which we used to categorize the year into three distinct periods (hereafter, seasons) of high (March–May), intermediate (June–August), and low (September–February) insect diversity (Fig. 1A; groups D, C, and A, respectively). These insect‐diversity‐based seasons closely follow normal annual temperature and precipitation trends for this area (Appendix S2: Fig. S1; Western Regional Climate Center 2018) and likely correspond with insects emerging after the rainy period (January–March) and then dying back as temperatures rise in July and August. As we were specifically interested in the role differences in local environmental variables on the differences in insect diversity and seasonal variation far outweighed local differences, we examined how differences in insect species richness, abundances, and species composition were affected by urbanization and site‐specific variables within each season described above.

To address differences in insect species richness and abundance within a season, we used the same repeated measures linear model approach we used for the whole year described above. We used post‐hoc Tukey's HSD tests for pairwise comparisons of urban types when significant differences were detected in the global model. Raw species richness and abundance values were first corrected for the number of days a trap was active at a site to control for differences in the collection time periods (i.e., raw values were divided by trap days). Corrected abundance values were then log‐transformed to conform to test assumptions of normality (confirmed with Shapiro‐Wilks tests).

### Species composition

We used PERMANOVA analyses (9,999 permutations) to explore how insect species composition changed across seasons, broad‐scale urbanization gradients (the seven urban types), and local habitat characteristics (six different local binary site descriptors). First, we created Bray‐Curtis resemblance matrices from square‐root‐transformed abundance values of individual species in a collection. To test for seasonal differences in insect species composition, we performed PERMANOVA on the whole year, treating seasons as fixed effects and months within a season and sites as random grouping factors to account for repeated measurements at sites (Anderson et al. 2008). To test for effects of urbanization and local habitat characteristics, we performed individual tests within season where we treated urban types and the six binary site descriptors as fixed effects and months and sites as random effects. We used post‐hoc pairwise PERMANOVA to explore differences in the specific urban types when global tests were significant. We also used indicator species analyses to determine which taxa were most responsible for any differences detected across the three seasons, seven urban types, and the binary site descriptors (Dufrêne and Legendre 1997, de Cáceres and Legendre 2009). We reduced the community to only those species that showed up in >5% of collections so as to focus on species most likely to contribute to large compositional differences among sites (81 species).

We used permutation‐based Mantel tests (Borcard and Legendre 2012; 9,999 permutations) to determine if insect species composition could be predicted by the local environmental parameters we calculated from the weather station measurements. We used the same species composition Bray‐Curtis resemblance matrices discussed above for analyses. All seven environmental measurements within a season were standardized before creating Euclidean distance matrices of environmental variables for each season. Where Mantel tests indicated a significant relationship between species composition and the environment, we used vector fitting to determine the relative contribution of each environmental variable to overall species composition.

We visualized the effects of urban types and the binary site descriptors on species composition using non‐metric multidimensional scaling (NMDS, 9,999 permutations, three dimensions) and fit significant environmental variables as vectors to the ordination.

We used PERMDISP analyses (Anderson et al. 2006, 2011) to explore how our landscape‐scale urbanization types and local‐scale binary site characteristics influenced local beta diversity. PERMDISP compares the average distance of individual samples within a fixed‐factor‐defined sample group to a group‐defined centroid in multivariate space created from a similarity matrix. Higher beta diversity groups have larger average distances among sites from the group‐defined centroid (i.e., are more dispersed). We used the same Bray‐Curtis resemblance matrices used for PERMANOVA in these analyses.

Species accumulation curves were calculated using EstimateS version 9.1.0 (Colwell 2009). Linear models, Mantel tests, indicator species analyses, and vector fitting were conducted in the R statistical package version 3.5.0 (R Core Team 2018) including packages indicspecies, nlme, and vegan (de Cáceres and Legendre 2009, Oksanen et al. 2013, Pinheiro et al. 2014). PERMANOVA and PERMDISP were conducted in PRIMER version 6.1.18 including the PERMANOVA+ package 1.0.8 (Anderson et al. 2008).

## Results

A total of 53,199 individual insects were identified from 360 collections taken at 30 sites across Los Angeles County from January through December 2014. We identified 217 species or morphospecies in 18 families across the three targeted orders of insects. Species accumulation curves reached asymptotes for all seven focal taxa (e.g., five families of flies, bees, and macrolepidoptera; Appendix S2: Fig. S3). Comparisons against predicted maxima indicated that the samples captured >70% of expected species with all but one group (bees) exceeding 85% coverage (Appendix S2: Table S2). *Megaselia agarici* (Lintner) (Diptera: Phoridae) was the most abundant and common insect found in the Malaise traps with 10,890 individuals occurring across 94% of collections. Seven other species also occurred in >50% of collections (five phorid flies and two bees). A total of 62 species (29% of all species) occurred in fewer than 1% of collections (Appendix S2: Table S3). Patterns of insect occurrence were only significantly predicted by spatial autocorrelation among collection sites during a single collection period (March) suggesting spatial autocorrelation is rare and likely unimportant in this system at the scale of this project. This corroborates previous findings of limited spatial patterns in insect diversity in the urban center of Los Angeles (McGlynn et al. 2019).

### Species richness and abundance

**Figure 2:**
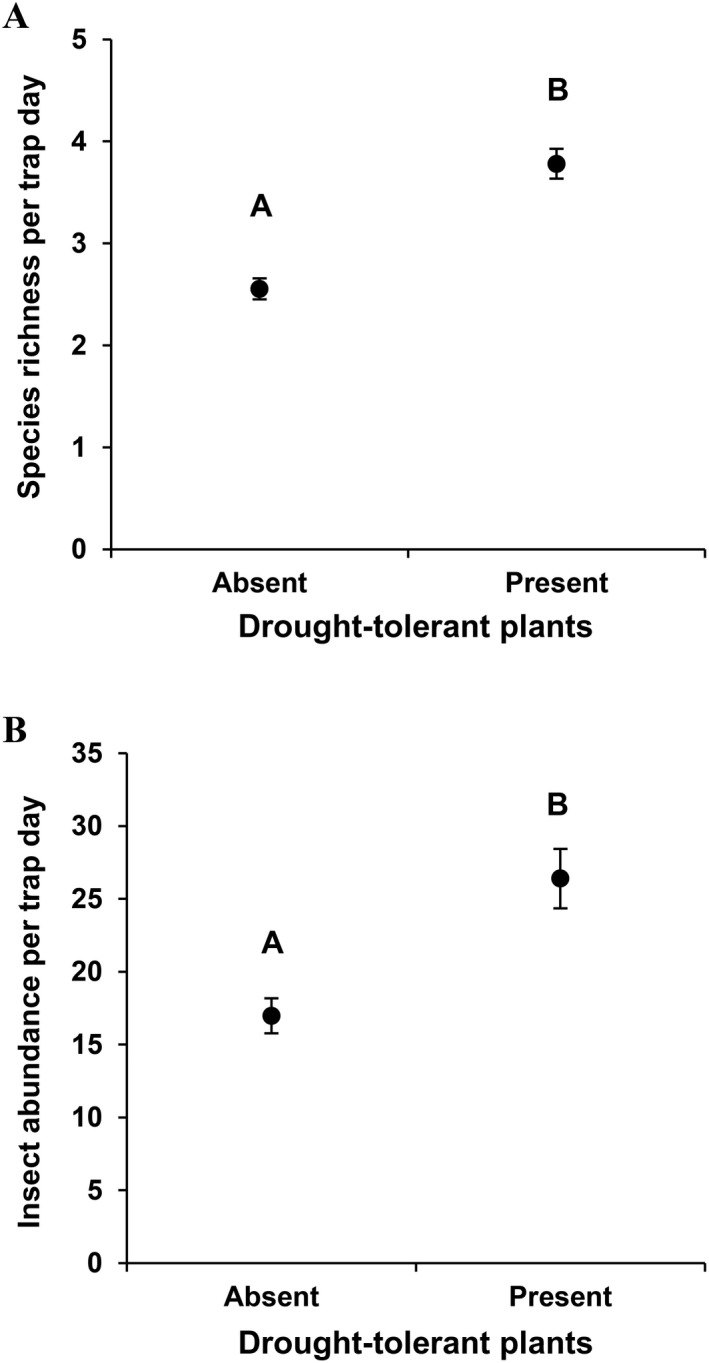
Raw (A) insect species richness and (B) insect abundance per trap day in sites in which drought‐tolerant plants were present across all 12 months of the study. Values are mean ± SE. Different letters indicate significant difference in means (P≤ 0.05). This pattern also was similar across the individual

At the time scale of a whole year, difference between local environmental parameters across months (e.g., normal seasonal differences in weather; Appendix S2: Tables S4 and S5; *F*_1, 313_ > 27.41, *P* < 0.0001 for month × PCA1 and month × PCA2) was the major driver of change in insect species richness and abundance. Specifically, insect species richness and abundance varied by month in a roughly seasonal pattern marked by a peak occurring from March through May, a decline from June through August, and a period of lower diversity from September through February (*F*_11, 319_ > 12.25, *P* < 0.0001 for both richness and abundance tests; Fig. 1). Other than normal seasonal variation, the presence of drought‐tolerant plants also was associated with 48% higher insect species richness and 55% higher insect abundance at a site throughout the year (Fig. 2).

**Figure 3:**
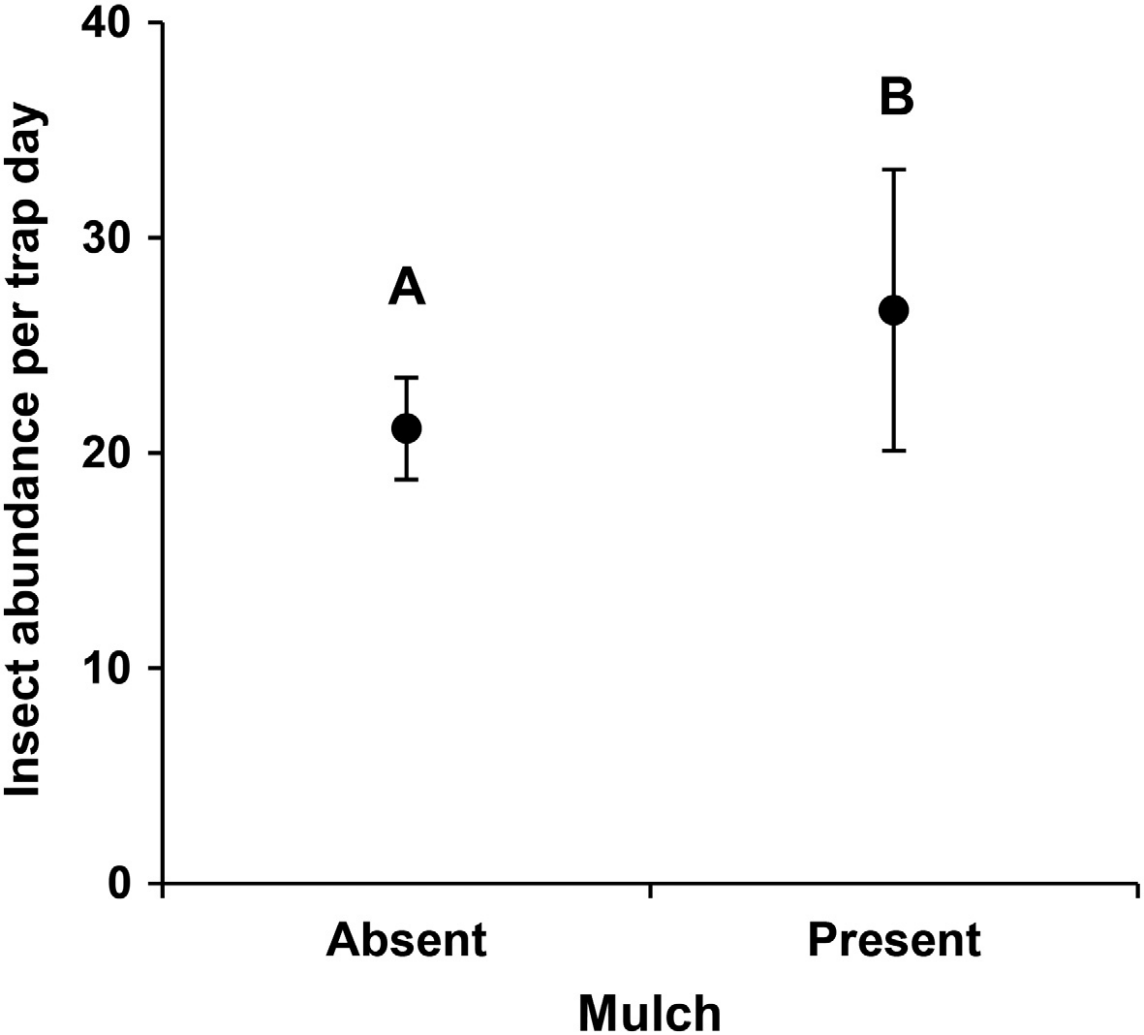
Raw insect abundance per trap day in sites with and without mulch. Only collections made during June through August are included.Values are mean ± SE. Different letters indicate significant difference in means (P ≤.05).

When exploring the drivers of insect diversity within a season, the relative importance of local‐scale habitat differences and environmental parameters became more apparent. Specifically, both insect species richness and abundance were >30% higher in sites with drought‐tolerant plants compared to sites without such plants (*F*_1,17_ > 4.27, *P* < 0.05; Appendix S2: Tables S6–S11) in all three seasons. The presence of mulch also was associated with >25% higher insect abundance but this only occurred for collections made during the months of June through August (*F*_1,17_ = 5.97, *P* = 0.03; Fig. 3; Appendix S2: Table S11). No other local habitat variables we accounted for affected insect species richness or abundance.

**Figure 4:**
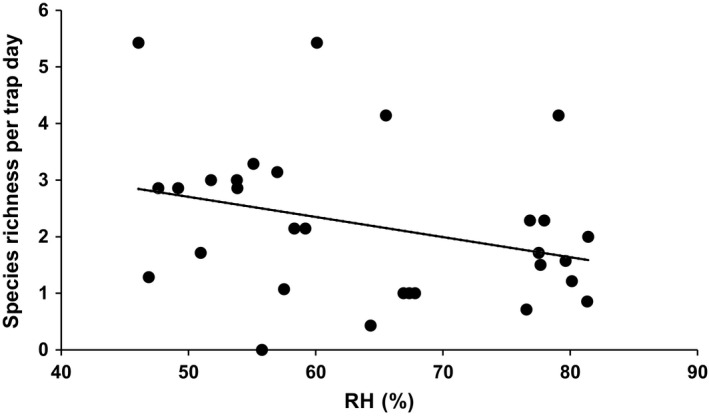
Insect species richness per trap day over daily mean relative humidity (RH) readings measured at highly urbanized sites (urban type 8) for collections made from September through February.

Local air temperature, relative humidity, and solar radiation (defined by PCA axes 1 and 2 in all models) had variable effects on insect communities depending on the specific season. For example, insect species richness increased as a product of an urban type by local environmental interaction during the low insect diversity season from September through February (*F*_6, 133_ = 2.18, *P* = 0.05; Appendix S2: Table S6). Specifically, species richness correlated with PCA2 scores but only in urban type 8 (*F*_1,24_ = 7.38, *P* = 0.01). PCA2 is best defined by a correlation with mean daily RH (Table 1) indicating that high insect species richness occurred in sites that were consistently drier (e.g., lower RH; Fig. 4). This correlation between insect species richness and RH was not significant in any of the other urban types during this time period. During March through May when richness peaked, insect abundance also decreased along PCA axis 2 (*F*_1,17_ = 6.91, *P* = 0.02; Appendix S2: Table S9) again indicating that the highest insect abundances occurred in relatively drier, hotter sites during this time period.

**Table 1:** The seven environmental variables input into the PCA and vector fitting analyses for the entire year of collections and for each of the three seasons within the year

Period and environmental variable	PCA1	PCA2	*R* ^2^	*P*
Year				
Maximum air temperature	0.48	−0.08	0.32	0.0001
Minimum air temperature	0.39	0.41	0.50	0.0001
Mean air temperature	0.49	0.25	0.56	0.0001
Maximum RH	−0.39	0.18	0.06	0.0002
Minimum RH	−0.18	0.60	0.06	0.0002
Mean RH	−0.31	0.53	0.02	0.0781
Photoperiod	0.31	0.31	0.23	0.0001
September–February				
Maximum air temperature	0.51	−0.21	0.58	0.0001
Minimum air temperature	0.36	−0.41	0.46	0.0001
Mean air temperature	0.50	−0.35	0.61	0.0001
Maximum RH	−0.34	−0.38	0.11	0.0003
Minimum RH	−0.26	−0.43	0.09	0.0011
Mean RH	−0.41	−0.54	0.14	0.0001
Photoperiod	0.15	−0.22	0.27	0.0001
March–May				
Maximum air temperature	0.36	0.46	‐	‐
Minimum air temperature	0.19	−0.33	‐	‐
Mean air temperature	0.25	0.05	‐	−
Maximum RH	−0.71	0.51	‐	‐
Minimum RH	−0.23	−0.53	‐	‐
Mean RH	−0.36	−0.36	‐	‐
Photoperiod	0.30	−0.05	‐	‐
June–August				
Maximum air temperature	−0.62	−0.02	‐	‐
Minimum air temperature	−0.34	0.47	‐	‐
Mean air temperature	−0.49	0.23	‐	‐
Maximum RH	0.07	−0.53	‐	‐
Minimum RH	0.44	0.62	‐	‐
Mean RH	0.25	0.10	‐	‐
Photoperiod	0.00	−0.21	‐	‐

*Notes:*

Included with each variable are the eigenvectors along PCA axis 1 and 2. When environmental variables significantly influenced species composition, the *R* ^2^ value and *P* value from the vector‐fitting analyses are also provided for each variable. The variation explained by PCA axes 1 and 2 is 45.8% and 32.0%; 61.7% and 25.7%; 71.1% and 12.7%; 50.6% and 29.6%, for the whole year, September–February collections, March–May collections, and June–August collections, respectively. RH, relative humidity.

The relative importance of the local environment to insect species richness and abundance was more complicated for collections made during the hottest part of the year from June through August. Specifically, insect species richness changed with differences in the local environment but only within June and August collections (month × PCA1 and month × PCA2 interactions; Appendix S2: Tables S10 and S11). Insect species richness negatively correlated with values from PCA axis 2 in July and PCA axis 1 in August (*F*_1,28_ > 3.89, *P* < 0.05). PCA1 correlates most strongly with decreasing temperature (Table 1) indicating that hotter sites tended to have slightly increased species richness in August (Appendix S2: Fig. S4). PCA axis 2 correlates negatively with maximum daily RH but positively with minimum daily RH (Table 1). These correlations translate to insect species richness being higher at sites that had consistently higher maximum RH (Appendix S2: Fig. S5a) but lower at sites that higher minimum RH (Appendix S2: Fig. S5b). Effectively, this means that during the hottest part of the year (June–August) insect species richness was highest in sites that were wet at some point during the collection week but that dry up and are not consistently wet throughout the collection period.

**Figure 5:**
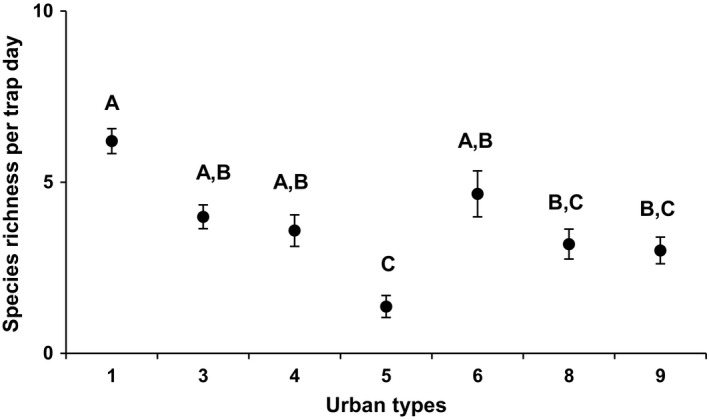
Raw insect species richness per trap day across the seven represented urban types for collections made from June through August. Values are mean ± SE. Different letters indicate significant difference in means (P ≤.05).

Landscape‐scale urbanization patterns had little effect on insect species richness and abundance. The only consistent patterns we detected occurred during collections made in June through August where insect species richness was typically higher in less urbanized locations compared to more urbanized locations (*F*_6,17_ = 3.15, *P* = 0.03; Fig. 5). Specifically, post hoc Tukey's HSD indicated that insect species richness at sites in urban type 1 was higher than collections from urban types 5, 8, and 9. Collections made in urban types 3 and 4 also had higher species richness than collections made in urban type 5.

Collectively, these results reveal that seasonal fluctuations in insect species richness and abundance have the greatest impact on local insect diversity throughout the year. After accounting for seasonal differences, local habitat and local microclimatic differences played important roles in shaping local insect communities. Specifically, sites with drought‐tolerant plants consistently had higher insect species richness and abundance across the urban matrix of Los Angeles while sites that were hotter and drier also were generally associated with a more diverse insect fauna. In contrast, landscape‐scale urbanization only decreased insect species richness during the June through August collections.

## Species composition

**Figure 6:**
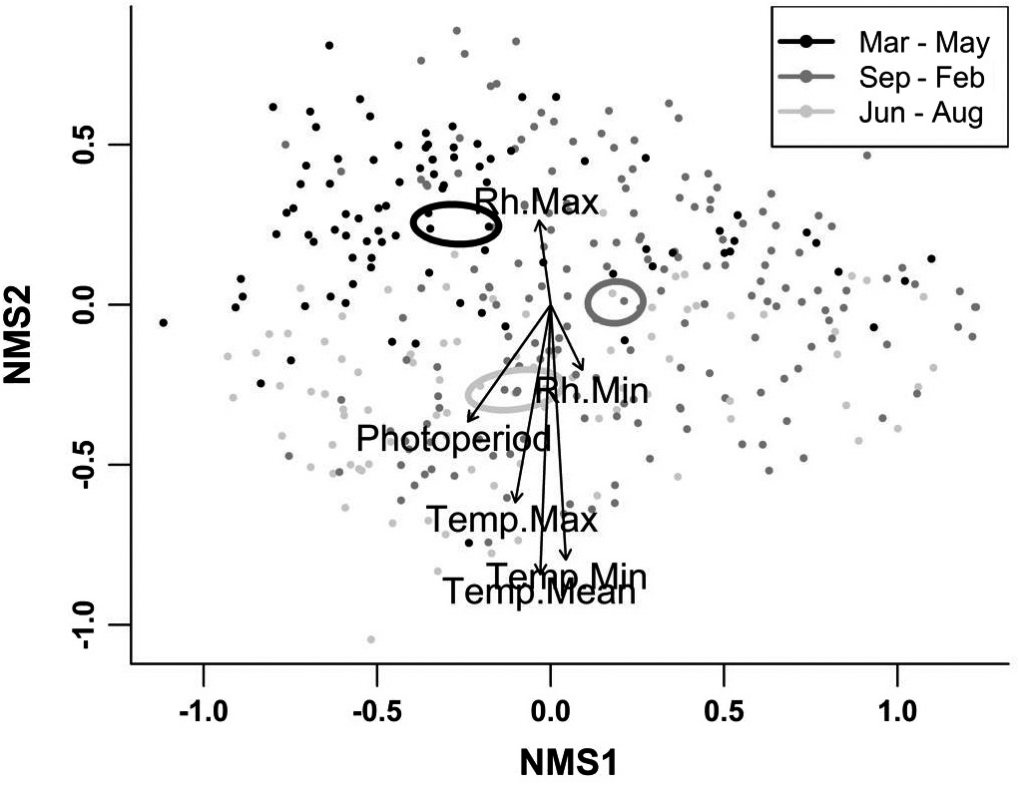
An ordination of species composition throughout all surveys along nonmetric mutidimensional scaling (NMDS) axis 1 and 2 (stress =0.16). These axes were chosen to best visualize differences among seasons and along environmental vectors. Points represent individual collections. Sites are colored by season. A legend matching colors with season is provided in the top right of the figure. Ellipses indicate the standard error measurements around the centroid of each season. Vectors indicate significant correlations between species composition and a measured environmental variable. The length of each vector is proportional to the strength of the correlation.

Insect species composition had clear seasonal trends and differed among the three seasons (pseudo‐*F*_2, 261_ = 2.49, *P* = 0.0001; Fig. 6). Cosmopolitan species such as many *Drosophila* and *Megaselia* species tended to occur most frequently during the March through May collections during peaks in insect abundance. Pollinators include several bee genera (*Apis*,* Halictus*,* Lasioglossum*, and *Megachile*) occurred most often in collections made from June through August. As with insect species richness and abundance, differences in insect species composition corresponded with normal seasonal increases in temperature (Mantel test, *r* = 0.12, *P* = 0.0001; Table 1).

Within seasons, insect species composition largely differed among sites as a product of landscape‐scale urbanization (pseudo‐*F* > 1.21, *P* < 0.03 for urban types in each season). Pairwise comparisons among urban types revealed that highly urbanized areas (urban type 5, 6, 8, and 9) differed from less urbanized areas (urban type 3 and 4) in collections from both June through August and September through February (*t* > 1.20, *P* < 0.05; Appendix S2: Tables S13a and S13c, Figs. S5 and S6). Pairwise comparisons of collections made from March through May also revealed differences in insect composition in some less urbanized and more urbanized areas (types 4 and 8; Appendix S2: Table S13b, Fig. S6) but also a differences in the insect community between some less urbanized sites (types 3 and 4). Indicator species revealed that more urbanized sites hosted more cosmopolitan fungivore and detritivore phorid fly species such as *Megaselia agarici*,* Megaselia rufipes*, and *Megaselia scalaris* (Appendix S2: Table S14a–S16a) but the number of new species and lack of information available for many of the known species of flies makes determining strong relationships between natural history strategies and location difficult (Hartop et al. 2015, Brown and Hartop 2017).

Local habitat differences also shaped local insect species composition. The presence of drought‐tolerant plants, native plants, and compost was frequently associated with differences in insect composition (pseudo‐*F* > 1.37, *P* < 0.05; Appendix S2: Fig. S5–S7). Sites with native and drought‐tolerant plants had a wide range of associated insect fauna and both included notable pollinators such as *Apis mellifera*, other bees (*Lasioglossum*¸ *Megachile*, and *Sphecodes*) and several flower flies (*Allograpta*,* Dioprosopa*, and *Toxomerus*); whereas sites without these plants had no indicator species, which suggests communities of insects were composed of fairly random assortments of insects from the larger regional species pool. Drosophilid flies tended to occur more frequently at sites that had compost present; whereas, a host of *Megaselia* phorid flies were more often found at sites without compost (Appendix S2: Table S14–S16).

Within a season, the local environmental variables measured generally had little effect on insect species compositions. Specifically, Mantel tests revealed no significant correlations between the environmental variables we measured and insect species in collections from March through May and from June through August (*r* < 0.07, *P* > 0.07). High daily temperatures did play a role in determining local species composition during the low seasons from September through February (Mantel test, *r* = 0.20, *P* < 0.0001; Table 1).

Collectively these results indicate that broad‐scale urbanization had a strong effect on insect species composition potentially promoting communities of urbanophile flies. The presence of native or drought‐tolerant plants and compost in a yard also promoted a more diverse insect community regardless of the broader impacts of urbanization. In contrast with measurements of insect species richness and abundance, the local microclimate did not generally influence insect species composition except during September through February collections. Turf lawns, mulched flowerbeds, and frequent watering did not affect insect species composition.

Beta diversity among sites within a given season was fairly low; however, we detected some differences during June through August collections. Specifically, during this period, sites that had native or drought‐tolerant plants tended to have more similar insect communities (lower beta diversity) compared to those sites that did not host native or drought‐tolerant plants (*F*_1,88_ > 6.95, *P* < 0.03). There were also differences in beta diversity among urban types during this period (*F*_6,83_ = 7.82, *P* = 0.0001). Urban types 1 and 6 had much lower beta diversity measurements compared to other types (Appendix S2: Table S17); however, these urban types also had very low samples sizes (*n* = 3 and 6, respectively), which strongly influence beta diversity measurements. Pairwise tests also revealed that beta diversity was lower in urban type 4 compared to both urban type 3 and urban type 5 (*t* > 2.51, *P* < 0.03). Finally, during the September through February collection, sites with compost often had more variable insect communities compared to sites without compost (*F*_1, 178_ = 6.09, *P* = 0.02).

## Discussion

Urban biodiversity must contend with both broad‐scale gradients of urbanization and highly heterogeneous local habitats (Pickett et al. 2017). Understanding multiscale effects on biodiversity is fundamental to ecological theory (Levin 1992) and necessary for informing future urban restoration (McDonnell and MacGregor‐Fors 2016), especially within urban biodiversity hot spots that likely host many unknown or poorly known species (Hartop et al. 2015, Brown and Hartop 2017). Here we show that insect communities in Los Angeles County are shaped by a combination of factors operating at both the local and landscape scales. Specifically, insect species richness and abundance were best predicted by fine‐scale variables such as local temperature, local relative humidity, and the presence of drought‐tolerant plants. However, the magnitude of the negative effects of high relative humidity on insect richness changed with season and, to a lesser degree, increased urbanization. In contrast to species richness, differences in local insect species composition were best explained by broader categories of urbanization with more diverse and native insect assemblages occurring in less urbanized areas. Importantly, the presence of drought‐tolerant plants was associated with more diverse insect assemblages that exhibited low beta diversity among sites and hosted many important pollinator species, regardless of urbanization. These results, along with other recent studies of urban biodiversity (Kyrö et al. 2018, Anderson et al. 2019, Burdine and McCluney 2019) show that the interaction between broad‐scale urban gradients and fine‐scale habitat variables contribute to the overall community structure in urban ecosystem and must be considered when developing management practices intended to enhance the biodiversity of urbanized landscapes.

Our finding that hotter, drier sites have higher insect abundance differs from the findings in Phoenix, Arizona, USA (Bang and Faeth 2011). In Phoenix, the highest abundances of ground‐dwelling arthropods occurred in well‐watered residential yards (Bang and Faeth 2011) and irrigated agricultural sites (Cook and Faeth 2006). However, the work conducted in Phoenix also showed that some insect taxa (e.g., ground beetles) were more abundant in drier sites. Our study indicates that sites more closely emulating the natural environment (e.g., drier with drought‐tolerant, native flora) support a wider array of insects in Los Angeles. It is important to note that the collection techniques differ between these two projects (pitfall traps vs. Malaise traps), thus confounding comparisons to some degree. However, these collection techniques are fairly taxon‐specific (Townes 1972, Work et al. 2002)—pitfall traps target ground‐dwelling species while Malaise traps generally target species that rely more heavily on flight—and differences between the results likely reflect real differences in the drivers of diversity for these insect groups. These differences also highlight the need for standardized, multi‐city studies of urban biodiversity (Parker 2015, Groffman et al. 2017; e.g., Barbato et al. 2017, Youngsteadt et al. 2017, Anderson et al. 2019) comparable to what is seen in global studies of other ecosystems (Condit 1998, Fraser et al. 2013, Anderson‐Teixeira et al. 2015). Insects and other arthropods are a convenient group for multi‐city comparisons as they are highly diverse, generally unmanaged, easy to collect, and respond quickly and predictably to environmental changes (McIntyre 2000, Brown 2018).

Whether native plants play an important role in supporting urban insect diversity depends on the focal taxa. For example bees, adult butterflies, and wasps often rely on nonnative, locally adapted plants for alternative food sources (Shapiro 2002) and are generally unaffected by augmenting urban landscapes with native vegetation (Matteson and Langellotto 2011). In contrast, herbivorous insects such as caterpillars often require their native host plants to persist in the environment (Burghardt et al. 2009, Narango et al. 2017). By taking a community approach, we show that not only native plants but also climate‐appropriate, nonnative plants can positively impact insect communities. Regardless, planting native plants in urban landscapes should be a priority as native plants and their insect herbivores have disproportionately large impacts on the whole food chain (Narango et al. 2017, 2018) and native plants are intrinsically adapted to the regional environment.

There is a high degree of similarity in insect communities across the urban landscape of Los Angeles. This is especially true when holistically comparing collection throughout the whole year, as insect species composition among urban types frequently separates only during certain seasons. Similarity among urban types could be attributed to a few factors. First, the species composition of insect communities in the most urbanized sites (urban types 5–9) could converge due to introductions of dominant invasives or high abundance of cosmopolitan insect urbanophiles (Holway and Suarez 2006, Shochat et al. 2010, Faeth et al. 2011). Indeed, we observed a higher abundance of more cosmopolitan species like *Megaselia nigra* and *Megaselia scalaris* in urban types 5–9. Next, failure to detect differences among insect communities in less urbanized locations could be attributed to low representation of certain habitat types among our study sites. Specifically, urban type 2 (wetlands) was not represented in this study and urban type 1 (low development with natural vegetation) was only represented by a single site. Although it was not statistically significant, urban type 1 generally separates from other urban types in NMDS ordinations and had the highest raw number of species and rare species of phorid flies (Brown and Hartop 2017). It is possible that increased replication would result in detecting differences in insect communities among less urbanized sites. In fact, other work in this system detected different compositions of common plants and animals among the four less urbanized typologies using iNaturalist data (Li et al. 2019).

By pairing site‐specific measurements within a broad‐scale urban classification scheme that considers both biophysical and anthropogenic variables, we are able to determine three multiscale drivers of insect diversity across Los Angeles County: seasonality, broad‐scale urban gradient, and local landscaping. Our findings indicate that land managers, city planners, and individual property owners can increase local biodiversity by planting climate‐appropriate or native flora and by maintaining landscapes that more closely match conditions in nearby natural areas. Indeed, the simple binary description highlights the profound effect that drought‐tolerant and native plants have on insect populations in Los Angeles. Planting native or climate‐appropriate plants could help ameliorate biotic homogenization in cases where it results from the loss of native habitat (McKinney 2006, Wheeler et al. 2017), an especially important consideration in cities like Los Angeles that are situated in biodiversity hot spots like the California Floristic Provence and contribute substantially to habitat loss due to urbanization. Finally, our study demonstrates that urban community assembly is complex, operates across multiple scales, and deserves future study across a broader range of cities with variable background climates and habitat types.

## Data Availability

Data are available from the Dryad Digital Repository: https://doi.org/10.5061/dryad.7d7wm37rd
