# Supplementary material for: Local‐ and landscape‐scale variables shape insect diversity in an urban biodiversity hot spot
Source: Ecol Appl. 2020 Mar 5;30(4):e02089. doi: 10.1002/eap.2089 (PMC7317463; doi:10.1002/eap.2089)
Supplement: Supplementary file 1 [file EAP-30-e02089-s001.pdf]

Supporting Information. Adams, B. J., E. Li, C. A. Bahlai, E. K. Meineke, T. P McGlynn, and B. V. Brown. 2020. Local and landscape scale variables shape insect diversity in an urban biodiversity hotspot. Ecological Applications.

Appendix S1

The urban habitat classification is designed specifically to understand broad-scale urbanization patterns across Los Angeles county. Eighteen ecological and anthropogenic factors were selected for creating this typology based on their effects on urban biodiversity and data availability and accessibility in the Los Angeles County. In general, they fall into three categories: the biophysical environment (including, average annual temperature, maximum temperature, average annual precipitation, elevation, slope, percentage of forest, percentage of grassland, percentage of water and wetlands), the built environment(including, tree canopy, greenness, imperviousness, percentage of urban open space, percentage of urban areas, distance to the nearest natural areas), and the social environment(including, population, population density, traffic density, and traffic noise). For detailed variable selections and descriptions, see Li at al. (2019).

We used Block Groups as our geographic unit for habitat classification. Hierarchical cluster analysis was adopted to categorize the 6,040 Block Groups in the Los Angeles County into a manageable number of types based on variation in the 18 input variables. We used a combination of the dendrogram and gap statistic to identify the optimal number of clusters. All analyses were performed in R 3.4.2 utilizing fastcluster, dendextend, and tidyverse packages.

Our results indicated that our study area can be differentiated into substantively nine different types. Each type was given a name that reflects its geographic location and/or an additional distinctive feature to improve communication about the types (see Table S1).

Literature Citations

Li, E., S. S. Parker, G. B. Pauly, J. M. Randall, B. V. Brown, and B. C. Cohen. 2019. An urban biodiversity assessment framework that combines an urban habitat classification scheme and citizen science data. Frontiers in Ecology and Evolution 7:277.
