# Supplementary material for: Local‐ and landscape‐scale variables shape insect diversity in an urban biodiversity hot spot
Source: Ecol Appl. 2020 Mar 5;30(4):e02089. doi: 10.1002/eap.2089 (PMC7317463; doi:10.1002/eap.2089)
Supplement: Supplementary file 2 [file EAP-30-e02089-s002.pdf]

Supporting Information. Adams, B. J., E. Li, C. A. Bahlai, E. K. Meineke, T. P McGlynn, and B. V. Brown. 2020. Local and landscape scale variables shape insect diversity in an urban biodiversity hotspot. Ecological Applications.

Appendix S2

Tables

Table S1.

The descriptions of the nine urban habitat classification types developed using 18 continuous environmental variables. Types 1 through 4 represent the less urbanized areas and Types 5 through 9 represent more urbanized areas.

Urban Type	Description
**1**	**Low development with natural vegetation**
	· Very high natural vegetation, primary grassland and forest
	· Very steep terrain
	· Very low urban development and low population density
	· Relative high precipitation in the Los Angeles area
**2**	**Dams, reservoirs, and wetlands**
	· Low very vegetation cover
	· level terrain
	· Very low urban development
**3**	**Foothill areas**
	· Affluent and well vegetated neighborhoods
	· Primarily residential uses
	· Moderate terrain
	· Moderate building site coverage and population density
**4**	**Urban parks and open space**
	· High tree canopy coverage and green open space
	· Low urban development
	· Primarily parks, cemeteries, and golf courses
**5**	**Valley arterial areas**
	· Highest traffic density and traffic noise
	· High percentage of impervious surface

- Low in vegetation
- Primarily highways and surrounding neighborhoods

6 **Valley less developed areas**

- Moderate terrain, mostly in the San Fernando and San Gabriel Valleys
- Relatively hotter and wetter weather
- Low tree canopy coverage and greenness
- High urban development and population
- Primarily residential uses

7 **Basin less developed areas**

- Flat terrain, mostly in the Los Angeles Basin
- Relatively milder and dryer weather
- Low tree canopy coverage and greenness
- High urban development and population
- Primarily residential uses

8 **Most developed areas**

- Very high urban development and population
- Far from regional parks with natural vegetation
- Very low tree canopy coverage and greenness
- Mixed land use

9 **Furthest from regional parks with natural vegetation**

- High urban development and population
- Very high traffic density and noise
- Low tree canopy coverage and greenness
- Furthest from regional parks with natural vegetation
- Mixed land use

Table S2.

The total morphospecies with S_{est} and Chao1 estimators of maximum number of species and estimated percent community coverage for each of the seven focal taxa across the 360 samples represented in this study.

**Taxa**	**Morphospecies**	**S_{est}**	**Chao1**	**Coverage**
Bees	25	30.04	33.98	73%
Lepidoptera	24	25.37	24.50	95%
Drosophildae	22	23.57	22.66	93%
Phoridae	99	102.84	102.99	96%
Scatopsidae	7	7.00	7.00	100%
Syrphidae	29	32.67	32.99	88%
Tipulidae	11	12.87	12.00	85%

Table S3.

A list of all 217 morphospecies identified from the 360 collections including the percent of collections each morphospecies was found in (Total) and the percent of collections across each represented urban type (T1 – T9, excluding T2 and T7).

**Order**	**Family**	**Morphospecies + Authority**	**Total**	**T 1**	**T 3**	**T 4**	**T 5**	**T 6**	**T 8**	**T 9**
Diptera	Drosophilidae	*Amiota picta* (Coquillett 1904)	0.6	0.0	1.2	0.0	0.0	4.2	0.0	0.0
		Chymomyza amoena (Loew 1862)	0.6	0.0	1.2	0.0	0.0	4.2	0.0	0.0
		Drosophila busckii Coquillett 1901	15.3	8.3	15.5	4.2	16.7	16.7	28.3	11.9
		Drosophila flavohirta Malloch 1924	1.7	0.0	0.0	0.0	0.0	0.0	5.0	3.6
		Drosophila gentica Wheeler & Takada 1962	12.8	0.0	4.8	10.4	10.4	4.2	23.3	20.2
		Drosophila immigrans Sturtevant 1921	20.3	8.3	26.2	8.3	16.7	25.0	31.7	15.5
		Drosophila melanogaster Meigen 1830	2.2	0.0	2.4	0.0	0.0	0.0	10.0	0.0
		Drosophila occidentalis Spencer 1942	1.9	16.7	1.2	2.1	2.1	0.0	1.7	1.2
		Drosophila simulans Sturtevant 1919	15.3	0.0	21.4	8.3	10.4	12.5	21.7	14.3
		Drosophila sp1	16.9	8.3	20.2	4.2	6.3	25.0	31.7	15.5
		Drosophila sp2	2.5	0.0	1.2	2.1	0.0	4.2	6.7	2.4
		Drosophila suzukii (Matsumura 1931)	6.7	0.0	9.5	0.0	4.2	4.2	10.0	8.3
		Gitona americana Patterson 1943	10.6	25.0	27.4	2.1	0.0	20.8	3.3	4.8
		Gitona bivisualis Patterson 1943	2.2	16.7	6.0	0.0	0.0	0.0	0.0	1.2
		Repleta sp1	19.2	25.0	46.4	2.1	16.7	29.2	8.3	7.1
		Scaptomyza adusta Loew 1862	0.6	0.0	0.0	4.2	0.0	0.0	0.0	0.0
		Scaptomyza flava (Fallén 1823)	0.8	0.0	0.0	0.0	0.0	0.0	1.7	2.4
		Scaptomyza graminum (Fallén 1823)	0.8	0.0	1.2	0.0	2.1	0.0	1.7	0.0
		Scaptomyza paradusta Wheeler 1952	1.1	0.0	2.4	0.0	0.0	0.0	3.3	0.0
		Scaptomyza paravittata Wheeler 1952	0.3	0.0	0.0	0.0	2.1	0.0	0.0	0.0
		Scaptomyza terminalis (Loew 1863)	4.7	0.0	3.6	0.0	6.3	4.2	5.0	8.3
		Scaptomyza vittata (Coquillett 1895)	0.3	0.0	0.0	2.1	0.0	0.0	0.0	0.0
	Phoridae	*Aenigmatias* sp1	0.8	25.0	0.0	0.0	0.0	0.0	0.0	0.0
		Anevrina sp1	10.0	33.3	19.0	16.7	8.3	16.7	0.0	0.0
		Apocephalus aquilonius Brown 2002	0.6	8.3	1.2	0.0	0.0	0.0	0.0	0.0
		Apocephalus grandipalpis Borgmeier 1925	0.3	8.3	0.0	0.0	0.0	0.0	0.0	0.0
		Beckerina sp1	15.6	16.7	14.3	16.7	10.4	20.8	23.3	11.9
		Beckerina sp2	0.8	16.7	0.0	2.1	0.0	0.0	0.0	0.0
		Chonocephalus bentacaisei (Santos Abreu 1921)	30.6	41.7	42.9	31.3	6.3	12.5	50.0	21.4
		Conicera aldrichi Brues 1904	1.7	8.3	3.6	4.2	0.0	0.0	0.0	0.0
		Conicera similis (Haliday 1833)	33.3	8.3	46.4	20.8	29.2	66.7	36.7	21.4
		Conicera tibialis Schmitz 1925	16.4	0.0	14.3	8.3	16.7	37.5	21.7	15.5
		Diplonevra setigera (Malloch 1914)	5.8	16.7	15.5	4.2	2.1	8.3	1.7	0.0
		Dohrniphora cornuta (Bigot 1857)	23.1	8.3	23.8	10.4	14.6	29.2	43.3	20.2

Gymnophora sp1	0.6	16.7	0.0	0.0	0.0	0.0	0.0	0.0
Megaselia agarici (Lintner 1895)	91.7	100.0	91.7	100.0	85.4	100.0	88.3	89.3
Megaselia albicaudata Wood 1910	32.2	58.3	20.2	68.8	4.2	45.8	18.3	41.7
Megaselia arizonensis Malloch 1912	16.9	50.0	21.4	25.0	14.6	29.2	6.7	8.3
Megaselia armstrongorum Hartop et al. 2015	46.9	75.0	36.9	45.8	45.8	58.3	43.3	53.6
Megaselia atrox Borgmeier 1968	18.1	33.3	28.6	20.8	10.4	29.2	10.0	10.7
Megaselia baileyae Hartop et al. 2016	1.1	33.3	0.0	0.0	0.0	0.0	0.0	0.0
Megaselia barberi (Malloch 1912)	26.7	33.3	34.5	35.4	10.4	8.3	18.3	33.3
Megaselia basispinata Lundbeck 1920	21.4	8.3	17.9	10.4	8.3	33.3	35.0	27.4
Megaselia berndseni (Schmitz 1919)	31.9	41.7	40.5	52.1	14.6	58.3	13.3	26.2
Megaselia bradyi Hartop et al. 2015	0.3	0.0	0.0	2.1	0.0	0.0	0.0	0.0
Megaselia brejchaorum Hartop et al. 2015	2.8	8.3	6.0	4.2	0.0	0.0	1.7	1.2
Megaselia carthayensis Hartop et al. 2015	20.8	41.7	20.2	29.2	10.4	45.8	10.0	20.2
Megaselia cavifemur Borgmeier 1964	0.8	16.7	1.2	0.0	0.0	0.0	0.0	0.0
Megaselia ciancii Hartop et al. 2015	2.8	0.0	9.5	2.1	2.1	0.0	0.0	0.0
Megaselia creasoni Hartop et al. 2015	28.3	33.3	23.8	16.7	25.0	37.5	41.7	28.6
Megaselia defibaughorum Hartop et al. 2015	2.2	8.3	2.4	0.0	0.0	4.2	3.3	2.4
Megaselia donahuei Hartop et al. 2015	17.8	8.3	23.8	6.3	8.3	37.5	35.0	7.1
Megaselia francoae Hartop et al. 2015	24.7	50.0	25.0	33.3	31.3	29.2	21.7	13.1
Megaselia friedrichae Hartop et al. 2016	1.4	16.7	3.6	0.0	0.0	0.0	0.0	0.0
Megaselia fujiokai Hartop et al. 2015	24.4	41.7	22.6	31.3	10.4	8.3	23.3	33.3
Megaselia glabrifrons (Wood 1909)	0.8	16.7	0.0	0.0	0.0	0.0	0.0	1.2
Megaselia globipyga Borgmeier 1966	1.4	0.0	0.0	6.3	0.0	0.0	1.7	1.2
Megaselia gonzalezorum Hartop et al. 2016	1.1	0.0	0.0	2.1	0.0	4.2	3.3	0.0
Megaselia halterata (Wood 1910)	56.4	66.7	65.5	39.6	35.4	83.3	56.7	59.5
Megaselia hansonix Disney et al. 2009	26.1	41.7	32.1	16.7	16.7	25.0	38.3	20.2
Megaselia hardingorum Hartop et al. 2015	5.0	16.7	13.1	4.2	0.0	8.3	1.7	0.0
Megaselia heini Hartop et al. 2015	20.0	75.0	32.1	6.3	4.2	25.0	21.7	14.3
Megaselia hentschkeae Hartop et al. 2015	6.7	8.3	1.2	4.2	6.3	8.3	11.7	9.5
Megaselia hirticaudata Wood 1910	6.1	33.3	6.0	8.3	6.3	12.5	3.3	1.2
Megaselia hoffmanorum Hartop et al. 2015	0.6	8.3	1.2	0.0	0.0	0.0	0.0	0.0
Megaselia hoggorum Hartop et al. 2015	6.9	25.0	22.6	6.3	0.0	0.0	0.0	0.0
Megaselia hoguei Hartop et al. 2015	4.2	41.7	6.0	10.4	0.0	0.0	0.0	0.0
Megaselia ilca Borgmeier 1964	5.0	0.0	1.2	0.0	2.1	20.8	13.3	3.6
Megaselia isaacmajorum Hartop et al. 2015	2.2	41.7	1.2	4.2	0.0	0.0	0.0	0.0
Megaselia joanneae Hartop et al. 2016	1.1	8.3	2.4	0.0	0.0	0.0	1.7	0.0
Megaselia kelleri Hartop et al. 2015	16.9	8.3	8.3	25.0	4.2	29.2	16.7	26.2
Megaselia largifrontalis Schmitz 1939	36.9	83.3	59.5	47.9	22.9	45.8	26.7	14.3
Megaselia lombardorum Hartop et al. 2015	78.9	91.7	84.5	77.1	64.6	87.5	83.3	75.0
Megaselia losangelensis Hartop et al. 2016	1.4	0.0	1.2	0.0	2.1	8.3	0.0	1.2
Megaselia marquezi Hartop et al. 2015	44.4	25.0	36.9	39.6	18.8	54.2	66.7	53.6
Megaselia mikejohnsoni Hartop et al. 2015	23.6	50.0	22.6	25.0	14.6	29.2	18.3	27.4
Megaselia modesta (Brues 1919)	3.3	16.7	3.6	4.2	4.2	4.2	0.0	2.4

Megaselia nigra Meigen 1830	60.6	75.0	69.0	54.2	41.7	79.2	60.0	59.5
Megaselia oxboroughae Hartop et al. 2015	29.7	25.0	29.8	41.7	18.8	66.7	26.7	21.4
Megaselia phyllissunae Hartop et al. 2016	0.8	8.3	2.4	0.0	0.0	0.0	0.0	0.0
Megaselia pisanoi Hartop et al. 2015	6.7	50.0	9.5	20.8	0.0	0.0	0.0	0.0
Megaselia pleuralis Wood 1909	30.0	50.0	33.3	25.0	8.3	29.2	30.0	39.3
Megaselia pongsaiae Hartop et al. 2016	0.8	0.0	1.2	0.0	0.0	4.2	0.0	1.2
Megaselia renwickorum Hartop et al. 2015	5.3	25.0	4.8	8.3	0.0	12.5	1.7	4.8
Megaselia risoria Hartop et al. 2016	0.3	0.0	0.0	0.0	0.0	0.0	1.7	0.0
Megaselia rodriguezorum Hartop et al. 2015	6.1	8.3	4.8	0.0	6.3	8.3	6.7	9.5
Megaselia ruficornis (Meigen 1830)	6.1	25.0	6.0	2.1	4.2	0.0	11.7	4.8
Megaselia rufipes (Meigen 1804)	18.3	50.0	14.3	10.4	14.6	20.8	35.0	11.9
Megaselia sacatelensis Hartop et al. 2015	23.1	8.3	6.0	29.2	8.3	25.0	38.3	35.7
Megaselia scalaris Loew 1866	10.3	0.0	8.3	0.0	6.3	16.7	28.3	7.1
Megaselia scutellaris (Wood 1909)	1.9	8.3	2.4	8.3	0.0	0.0	0.0	0.0
Megaselia seaverorum Hartop et al. 2015	0.6	16.7	0.0	0.0	0.0	0.0	0.0	0.0
Megaselia shatesae Hartop et al. 2016	0.6	0.0	0.0	0.0	0.0	0.0	0.0	2.4
Megaselia sidneyae Hartop et al. 2015	34.2	50.0	47.6	43.8	16.7	37.5	25.0	28.6
Megaselia sordida Zetterstedt 1838	4.7	0.0	7.1	8.3	4.2	8.3	1.7	2.4
Megaselia steptoeae Hartop et al. 2015	42.8	8.3	38.1	31.3	33.3	62.5	61.7	45.2
Megaselia stoakesi Hartop et al. 2016	0.3	0.0	0.0	2.1	0.0	0.0	0.0	0.0
Megaselia studentorum Hartop et al. 2016	0.3	0.0	0.0	0.0	2.1	0.0	0.0	0.0
Megaselia sulphurizona Borgmeier 1966	85.0	100.0	85.7	91.7	81.3	91.7	80.0	82.1
Megaselia tecticauda Borgmeier 1964	14.2	83.3	16.7	6.3	0.0	54.2	3.3	10.7
Megaselia voluntariorum Hartop et al. 2016	0.3	8.3	0.0	0.0	0.0	0.0	0.0	0.0
Megaselia wiegmanae Hartop et al. 2015	62.8	33.3	56.0	56.3	39.6	87.5	78.3	72.6
Megaselia wongae Hartop et al. 2016	6.1	16.7	9.5	6.3	4.2	25.0	0.0	1.2
Metopina sp1	15.8	25.0	10.7	16.7	4.2	16.7	15.0	26.2
Metopina sp2	0.8	25.0	0.0	0.0	0.0	0.0	0.0	0.0
Microselia sp1	2.2	58.3	0.0	0.0	0.0	0.0	1.7	0.0
Myriophora alexandiae Hash et al. 2015	0.3	0.0	1.2	0.0	0.0	0.0	0.0	0.0
Phalacrotophora halictorum (Melander & Brues 1903)	18.1	33.3	45.2	37.5	4.2	8.3	0.0	1.2
Phora coangustata Schmitz 1927	0.6	0.0	1.2	0.0	0.0	0.0	0.0	1.2
Phora cristipes Schmitz and Wirth 1954	0.3	8.3	0.0	0.0	0.0	0.0	0.0	0.0
Phora sp1	1.4	0.0	1.2	8.3	0.0	0.0	0.0	0.0
Pseudacteon amuletum Plowes et al. 2009	1.1	25.0	1.2	0.0	0.0	0.0	0.0	0.0
Pseudacteon californiensis Disney 1982	1.1	25.0	0.0	0.0	0.0	0.0	0.0	1.2
Pseudacteon crawfordi Coquillett 1907	0.6	16.7	0.0	0.0	0.0	0.0	0.0	0.0
Pseudacteon sp1	0.6	16.7	0.0	0.0	0.0	0.0	0.0	0.0
Puliciphora occidentalis (Melander & Brues 1903)	28.9	8.3	11.9	43.8	22.9	70.8	26.7	33.3
Spiniphora bergenstammi (Mik 1864)	10.0	16.7	2.4	8.3	4.2	0.0	21.7	15.5
Triphleba sp1	0.8	16.7	0.0	0.0	0.0	0.0	1.7	0.0
Trophodeinus arizonensis (Borgmeier 1963)	2.8	66.7	0.0	4.2	0.0	0.0	0.0	0.0
Trophodeinus furcatus (Borgmeier 1963)	11.7	50.0	3.6	56.3	2.1	4.2	1.7	3.6

	Veruanus boreotis (Borgmeier 1963)	0.8	25.0	0.0	0.0	0.0	0.0	0.0	0.0
Scatopsidae	*Abrhexosa grossa* Cook 1965	3.3	8.3	4.8	12.5	2.1	0.0	0.0	0.0
	Aztecatopse sp1	2.5	8.3	3.6	4.2	0.0	0.0	0.0	3.6
	Coboldia fuscipes (Meigen 1830)	7.2	8.3	9.5	0.0	8.3	8.3	15.0	2.4
	Psectrosciara sp1	3.6	0.0	1.2	8.3	2.1	0.0	3.3	6.0
	Quateiella quatei (Cook 1956)	31.1	16.6	46.4	27.1	29.2	33.3	23.3	26.2
	Scatopse notata (Linnaeus 1758)	0.6	0.0	1.2	0.0	0.0	0.0	1.7	0.0
	Swammerdamella marginata Cook 1956	38.1	58.3	33.3	25.0	27.1	75.0	38.3	42.9
Syrphidae	*Allograpta exotica* (Wiedemann 1830)	6.1	8.3	3.6	2.1	8.3	8.3	5.0	9.5
	Allograpta obliqua (Say 1823)	20.3	0.0	17.9	18.8	18.8	12.5	25.0	26.2
	Chrysotoxum sp1	0.3	0.0	0.0	0.0	0.0	4.2	0.0	0.0
	Copestylum fraudulentum (Williston 1891)	0.6	8.3	1.2	0.0	0.0	0.0	0.0	0.0
	Copestylum marginatum (Say 1829)	1.7	0.0	3.6	2.1	0.0	0.0	1.7	1.2
	Copestylum satur (Osten Sacken 1877)	1.4	0.0	3.6	4.2	0.0	0.0	0.0	0.0
	Dioprosopa clavata (Fabricius 1794)	40.0	66.7	42.9	41.7	22.9	41.7	41.7	40.5
	Eumerus funeralis Meigen 1822	3.9	0.0	3.6	4.2	0.0	0.0	8.3	4.8
	Eumerus narcissi Smith 1928	0.6	0.0	1.2	0.0	0.0	4.2	0.0	0.0
	Eupeodes fumipennis (Thomson 1869)	2.8	8.3	0.0	2.1	4.2	4.2	3.3	3.6
	Eupeodes luniger (Meigen 1822)	0.3	0.0	0.0	0.0	0.0	0.0	1.7	0.0
	Eupeodes pomus (Curran 1921)	0.8	8.3	0.0	0.0	0.0	0.0	1.7	1.2
	Eupeodes sp1	3.1	0.0	4.8	4.2	2.1	0.0	3.3	2.4
	Eupeodes volucris Osten Sacken 1877	8.6	16.7	11.9	10.4	8.3	16.7	1.7	6.0
	Fazia micrura (Osten Sacken 1877)	4.7	0.0	3.6	8.3	2.1	4.2	8.3	3.6
	Heringia sp1	1.1	0.0	0.0	2.1	0.0	0.0	1.7	2.4
	Nausigaster unimaculata Townsend 1897	1.7	0.0	7.1	0.0	0.0	0.0	0.0	0.0
	Ocyptamus lemur (Osten Sacken 1877)	1.1	0.0	2.4	2.1	2.1	0.0	0.0	0.0
	Orthonevra flukei (Sedman 1964)	1.4	0.0	1.2	0.0	0.0	0.0	3.3	2.4
	Paragus haemorrhous Meigen 1822	34.2	16.7	36.9	43.8	29.2	33.3	23.3	39.3
	Platycheirus obscurus (Say 1824)	4.4	8.3	6.0	2.1	0.0	4.2	8.3	3.6
	Pseudodoros clavatus (Fabricius 1794)	0.8	0.0	1.2	0.0	2.1	0.0	0.0	1.2
	Spaerophoira sulphuripes (Thomson 1869)	1.9	0.0	1.2	2.1	2.1	4.2	1.7	2.4
	Sphaerophoria contigua Macquart 1847	0.3	0.0	0.0	0.0	2.1	0.0	0.0	0.0
	Syritta pipiens (Linnaeus 1758)	4.2	0.0	2.4	0.0	0.0	0.0	16.7	3.6
	Syrphus opinator Osten Sacken 1877	1.1	0.0	1.2	4.2	0.0	0.0	1.7	0.0
	Toxomerus marginatus (Say 1823)	17.5	41.7	11.9	14.6	8.3	45.8	8.3	25.0
	Toxomerus occidentalis Curran 1922	2.5	0.0	1.2	10.4	0.0	0.0	0.0	3.6
	Trichopsomyia sp1	0.3	0.0	1.2	0.0	0.0	0.0	0.0	0.0
Tipulidae	*Erioptera distincta* Alexander 1912	0.3	0.0	0.0	0.0	0.0	0.0	0.0	1.2
	Erioptera pilipes (Fabricius 1787)	1.4	0.0	0.0	2.1	0.0	4.2	3.3	1.2
	Limonia brevivena (Osten Sacken 1969)	0.6	0.0	0.0	0.0	0.0	4.2	0.0	1.2
	Limonia guatemalensis (Alexander 1916)	3.1	0.0	2.4	6.3	2.1	8.3	3.3	1.2
	Limonia humidicola (Osten Sacken 1859)	0.6	0.0	0.0	4.2	0.0	0.0	0.0	0.0
	Nephrotoma suturalis wulpiana (Bergroth 1888)	14.4	16.7	25.0	12.5	4.2	45.8	3.3	9.5

		Tipula atrisumma Doane 1912	0.3	0.0	0.0	0.0	0.0	4.2	0.0	0.0
		Tipula capistrano Alexander 1946	0.8	0.0	1.2	2.1	0.0	4.2	0.0	0.0
		Tipula oleracea Linnaeus 1758	1.9	0.0	1.2	4.2	0.0	0.0	1.7	3.6
		Tipula planicornis Doane 1912	2.5	0.0	3.6	2.1	0.0	8.3	3.3	1.2
		Tipula praecisa Loew 1872	0.8	0.0	2.4	0.0	0.0	4.2	0.0	0.0
Hymenoptera	Andrenidae	*Andrena* sp1	1.9	8.3	1.2	6.3	4.2	0.0	0.0	0.0
		Perdita sp1	0.8	0.0	0.0	4.2	0.0	0.0	0.0	1.2
	Apidae	*Anthophora californica* Cresson 1869	3.3	8.3	10.7	4.2	0.0	0.0	0.0	0.0
		Anthophora sp1	0.3	0.0	1.2	0.0	0.0	0.0	0.0	0.0
		Anthophora urbana Cresson 1878	1.1	0.0	1.2	4.2	0.0	0.0	0.0	1.2
		Apis mellifera Linnaeus 1758	54.2	33.3	60.7	54.2	39.6	79.2	55.0	51.2
		Ceratina sp1	16.4	16.7	21.4	18.8	12.5	12.5	13.3	15.5
		Diadasia sp1	0.3	0.0	1.2	0.0	0.0	0.0	0.0	0.0
		Eucera sp1	0.3	0.0	0.0	2.1	0.0	0.0	0.0	0.0
		Mellisodes sp1	4.4	16.7	7.1	6.3	0.0	4.2	0.0	4.8
		Xylocopa tabaniformis Smith 1854	0.3	0.0	1.2	0.0	0.0	0.0	0.0	0.0
	Colletidae	*Colletes* sp1	0.6	0.0	1.2	0.0	0.0	0.0	0.0	1.2
		Hylaeus sp1	8.1	16.7	11.9	6.3	10.4	16.7	1.7	4.8
	Halictidae	*Agapostemon* sp1	2.2	0.0	2.4	2.1	0.0	4.2	0.0	4.8
		Conanthalictus sp1	0.6	8.3	0.0	2.1	0.0	0.0	0.0	0.0
		Halictus ligatus Say 1837	1.1	0.0	0.0	0.0	2.1	0.0	0.0	3.6
		Halictus tripartitus Cockerell 1895	19.2	25.0	29.8	27.1	12.5	33.3	6.7	11.9
		Lasioglossum sp1	53.9	66.7	61.9	47.9	39.6	62.5	43.3	60.7
		Lasioglossum sp2	2.5	0.0	4.8	0.0	2.1	16.7	0.0	0.0
		Sphecodes sp1	29.4	25.0	36.9	25.0	25.0	41.7	11.7	36.9
	Megachilidae	*Ashmeadiella* sp1	0.3	0.0	1.2	0.0	0.0	0.0	0.0	0.0
		Heriades sp1	3.1	0.0	6.0	8.3	2.1	0.0	0.0	1.2
		Hoplitus sp1	0.3	0.0	0.0	0.0	0.0	0.0	0.0	1.2
		Megachile sp1	8.1	0.0	7.1	4.2	10.4	8.3	11.7	8.3
		Osmia sp1	1.1	0.0	1.2	2.1	0.0	0.0	0.0	2.4
Lepidoptera	Geometridae	*Dichorda illustraria* (Hulst 1886)	4.2	0.0	3.6	4.2	2.1	0.0	11.7	2.4
		Pero behrensaria (Packard 1871)	2.5	0.0	2.4	2.1	0.0	16.7	0.0	2.4
	Hesperiidea	*Erynnis funeralis* (Scudder & Burgess 1870)	3.3	8.3	2.4	0.0	0.0	4.2	8.3	3.6
		Hylephila phyleus (Drury 1773)	1.7	0.0	1.2	6.3	0.0	4.2	0.0	1.2
		Lerodea eufala (Edwards 1869)	2.5	0.0	6.0	0.0	2.1	4.2	0.0	2.4
		Poanes melane (Edwards 1869)	1.1	0.0	0.0	6.3	0.0	4.2	0.0	0.0
		Pyrgus albescens Plötz 1884	0.6	0.0	1.2	0.0	0.0	0.0	1.7	0.0
	Lycaenidae	*Brephidium exile* (Boisduval 1852)	5.0	0.0	8.3	4.2	10.4	0.0	3.3	2.4
		Leptotes marina (Reakirt 1868)	0.6	0.0	1.2	0.0	2.1	0.0	0.0	0.0
		Strymon melinus (Hübner 1818)	1.1	0.0	3.6	0.0	0.0	0.0	0.0	1.2
	Noctuidae	*Autographa californica* (Speyer 1875)	9.2	0.0	6.0	6.3	10.4	20.8	16.7	6.0
		Megalographa biloba (Stephens 1830)	3.1	0.0	3.6	4.2	4.2	0.0	1.7	3.6
		Noctua pronuba (Linnaeus 1758)	1.1	0.0	1.2	2.1	0.0	0.0	0.0	2.4

	Trichoplusia ni (Hübner [1803])	0.3	0.0	0.0	0.0	0.0	4.2	0.0	0.0
Nymphalidae	*Agraulis vanillae* (Linnaeus 1758)	6.1	8.3	3.6	2.1	2.1	12.5	10.0	8.3
	Danaus plexippus (Linnaeus 1758)	1.9	0.0	0.0	10.4	2.1	0.0	0.0	1.2
Papilionidae	*Papilio cresphontes* Cramer 1777	1.4	0.0	1.2	2.1	0.0	4.2	0.0	2.4
	Papilio zelicaon Lucas 1852	2.2	0.0	1.2	4.2	0.0	0.0	0.0	6.0
Pieridae	*Colias eurytheme* Boisduval 1852	3.6	0.0	1.2	4.2	6.3	8.3	3.3	3.6
	Phoebis sennae (Linnaeus 1758)	0.3	0.0	0.0	2.1	0.0	0.0	0.0	0.0
	Pieris rapae (Linnaeus 1758)	1.4	0.0	0.0	4.2	2.1	0.0	3.3	0.0
	Pontia protodice (Boisduval & Leconte [1830])	0.6	0.0	1.2	0.0	2.1	0.0	0.0	0.0
Sphingidae	*Hyles lineata* (Fabricius 1775)	3.3	0.0	1.2	8.3	4.2	12.5	0.0	2.4
	Manduca sexta (Linnaeus 1763)	0.6	0.0	0.0	0.0	0.0	0.0	0.0	2.4

Table S4.

Full model output from repeated measures linear model of insect species richness for the full year. Included in the table are the fixed effect terms from the model, the F-statistic with degrees of freedom, the p-value from partial F-tests, the model parameter, and the model estimates for each model parameter. Significant effects are bolded.

Fixed Effects	F	p-value	Model Parameters	coefficients (±SE)
**(Intercept)**	**$220.25_{(1,313)}$**	**<0.0001**	(Intercept)	6.41±1.26
**PCA1**	**$26.84_{(1,313)}$**	**<0.0001**	PCA1	1.37±0.21
PCA2	$0.02_{(1,313)}$	0.8985	PCA2	1.13±0.24
**Month**	**$12.7_{(1,313)}$**	**0.0004**	Month	-0.26±0.03
Compost	$0.08_{(1,17)}$	0.7823	Compost Present	-0.45±0.49
Mulch	$1.86_{(1,17)}$	0.1900	Mulch Present	1.17±0.69
**Drought Tolerant**	**$7.23_{(1,17)}$**	**0.0155**	Drought Tolerant Present	1.22±1.22
Native	$0.11_{(1,17)}$	0.7420	Native Present	-0.45±1.22
Lawn	$0.18_{(1,17)}$	0.6805	Lawn Present	-0.22±0.57
Water	$0.92_{(1,17)}$	0.3519	Water Frequent	-0.69±0.67
Urban Type	$1.47_{(6,17)}$	0.2464	Urban Type 3	-1.34±1.23
			Urban Type 4	-1.02±1.32
			Urban Type 5	-3.35±1.39
			Urban Type 6	0.45±1.5
			Urban Type 8	-1.67±1.28
			Urban Type 9	-1.32±1.25
Urban Type:PCA1	$1.65_{(6,313)}$	0.1339	Urban Type 3:PCA1	-0.52±0.21
			Urban Type 4:PCA1	-0.44±0.22
			Urban Type 5:PCA1	-0.73±0.22
			Urban Type 6:PCA1	-0.39±0.23
			Urban Type 8:PCA1	-0.43±0.21
			Urban Type 9:PCA1	-0.48±0.21
Urban Type:PCA2	$2.15_{(6,313)}$	0.0579	Urban Type 3:PCA2	-0.43±0.24
			Urban Type 4:PCA2	-0.5±0.26
			Urban Type 5:PCA2	-0.71±0.25
			Urban Type 6:PCA2	-0.4±0.28
			Urban Type 8:PCA2	-0.68±0.25
			Urban Type 9:PCA2	-0.57±0.24
**Month:PCA1**	**$28.44_{(1,313)}$**	**<0.0001**	Month:PCA1	-0.09±0.01
**Month:PCA2**	**$31.01_{(1,313)}$**	**<0.0001**	Month:PCA2	-0.08±0.01

Table S5.

Full model output from repeated measures linear model of insect abundance for the full year. Significant effects are bolded.

Fixed Effects	F	p-value	Model Parameters	coefficients (±SE)
**(Intercept)**	**399.42** $_{\mathbf{(1,313)}}$	**<0.0001**	(Intercept)	1.8±0.36
**PCA1**	**10.56** $_{\mathbf{(1,313)}}$	**0.0013**	PCA1	0.27±0.06
PCA2	$0.32_{(1,313)}$	0.5707	PCA2	0.26±0.07
Month	$0.24_{(1,313)}$	0.6225	Month	-0.06±0.01
Compost	$1.06_{(1,17)}$	0.3175	Compost Present	-0.22±0.14
Mulch	$1.92_{(1,17)}$	0.1841	Mulch Present	0.37±0.19
**Drought Tolerant**	**4.64** $_{\mathbf{(1,17)}}$	**0.0458**	Drought Tolerant Present	0.2±0.35
Native	$0.05_{(1,17)}$	0.8271	Native Present	-0.08±0.35
Lawn	$0.54_{(1,17)}$	0.4725	Lawn Present	-0.1±0.16
Water	$1.35_{(1,17)}$	0.2607	Water Frequent	-0.24±0.19
Urban Type	$1.11_{(6,17)}$	0.3991	Urban Type 3	-0.1±0.35
			Urban Type 4	-0.09±0.38
			Urban Type 5	-0.62±0.39
			Urban Type 6	0.52±0.42
			Urban Type 8	-0.24±0.36
			Urban Type 9	0.02±0.36
Urban Type:PCA1	$0.48_{(6,313)}$	0.8246	Urban Type 3:PCA1	-0.03±0.06
			Urban Type 4:PCA1	-0.01±0.06
			Urban Type 5:PCA1	-0.09±0.06
			Urban Type 6:PCA1	-0.04±0.07
			Urban Type 8:PCA1	-0.04±0.06
			Urban Type 9:PCA1	-0.06±0.06
Urban Type:PCA2	$1.98_{(6,313)}$	0.0679	Urban Type 3:PCA2	-0.08±0.07
			Urban Type 4:PCA2	-0.11±0.07
			Urban Type 5:PCA2	-0.19±0.07
			Urban Type 6:PCA2	-0.08±0.08
			Urban Type 8:PCA2	-0.15±0.07
			Urban Type 9:PCA2	-0.12±0.07
**Month:PCA1**	**39.03** $_{\mathbf{(1,313)}}$	**<0.0001**	Month:PCA1	-0.03±0
**Month:PCA2**	**27.41** $_{\mathbf{(1,313)}}$	**<0.0001**	Month:PCA2	-0.02±0

Table S6.

Full model output from repeated measures linear model of insect species richness for the low insect diversity season (September – February). Significant effects are bolded.

Fixed Effects	F	p-value	Model Parameters	coefficients (±SE)
**(Intercept)**	**$150.37_{(1,133)}$**	**<0.0001**	(Intercept)	3.9±1.37
PCA1	$3.05_{(1,133)}$	0.083	PCA1	0.72±0.3
PCA2	$0.64_{(1,133)}$	0.4238	PCA2	0.76±0.3
Month	$0.5_{(1,133)}$	0.4818	Month	-0.02±0.07
Compost	$0.09_{(1,17)}$	0.7678	Compost Present	-0.33±0.47
Mulch	$0.8_{(1,17)}$	0.3839	Mulch Present	0.55±0.66
**Drought Tolerant**	**$5.18_{(1,17)}$**	**0.0361**	Drought Tolerant Present	1.31±1.17
Native	$0.17_{(1,17)}$	0.6896	Native Present	-0.52±1.17
Lawn	$0.03_{(1,17)}$	0.8672	Lawn Present	-0.11±0.54
Water	$0.06_{(1,17)}$	0.8044	Water Frequent	-0.19±0.64
Urban Type	$0.82_{(6,17)}$	0.5696	Urban Type 3	-1.53±1.21
			Urban Type 4	-1.52±1.3
			Urban Type 5	-2.65±1.36
			Urban Type 6	-0.17±1.47
			Urban Type 8	-1.6±1.26
			Urban Type 9	-1.45±1.24
Urban Type:PCA1	$1.15_{(6,133)}$	0.3393	Urban Type 3:PCA1	-0.65±0.22
			Urban Type 4:PCA1	-0.6±0.23
			Urban Type 5:PCA1	-0.74±0.23
			Urban Type 6:PCA1	-0.54±0.24
			Urban Type 8:PCA1	-0.56±0.22
			Urban Type 9:PCA1	-0.56±0.22
**Urban Type:PCA2**	**$2.18_{(6,133)}$**	**0.0486**	Urban Type 3:PCA2	-0.66±0.27
			Urban Type 4:PCA2	-0.8±0.28
			Urban Type 5:PCA2	-0.69±0.28
			Urban Type 6:PCA2	-0.58±0.3
			Urban Type 8:PCA2	-0.93±0.28
			Urban Type 9:PCA2	-0.68±0.27
Month:PCA1	$0.41_{(1,133)}$	0.5233	Month:PCA1	-0.01±0.02
Month:PCA2	$0.59_{(1,133)}$	0.4421	Month:PCA2	-0.02±0.02

Table S7.

Full model output from repeated measures linear model of insect abundance for the low insect diversity season (September – February). Significant effects are bolded.

Fixed Effects	F	p-value	Model Parameters	coefficients (±SE)
**(Intercept)**	**$317.42_{(1,133)}$**	**<0.0001**	(Intercept)	1.11±0.42
PCA1	$1.31_{(1,133)}$	0.2553	PCA1	0.07±0.1
PCA2	$0.8_{(1,133)}$	0.3714	PCA2	0.12±0.1
**Month**	**$5.53_{(1,133)}$**	**0.0201**	Month	0.01±0.02
Compost	$1.01_{(1,17)}$	0.3299	Compost Present	-0.19±0.14
Mulch	$0.9_{(1,17)}$	0.3569	Mulch Present	0.25±0.19
**Drought Tolerant**	**$4.45_{(1,17)}$**	**0.0501**	Drought Tolerant Present	0.23±0.34
Native	$0.06_{(1,17)}$	0.8168	Native Present	-0.09±0.34
Lawn	$0.55_{(1,17)}$	0.4701	Lawn Present	-0.1±0.16
Water	$0.76_{(1,17)}$	0.3962	Water Frequent	-0.17±0.19
Urban Type	$1.16_{(6,17)}$	0.3704	Urban Type 3	-0.19±0.36
			Urban Type 4	-0.14±0.39
			Urban Type 5	-0.56±0.4
			Urban Type 6	0.45±0.44
			Urban Type 8	-0.24±0.38
			Urban Type 9	0.05±0.37
Urban Type:PCA1	$0.47_{(6,133)}$	0.8262	Urban Type 3:PCA1	-0.08±0.08
			Urban Type 4:PCA1	-0.04±0.08
			Urban Type 5:PCA1	-0.1±0.08
			Urban Type 6:PCA1	-0.06±0.09
			Urban Type 8:PCA1	-0.06±0.08
			Urban Type 9:PCA1	-0.06±0.08
Urban Type:PCA2	$0.86_{(6,133)}$	0.5298	Urban Type 3:PCA2	-0.13±0.09
			Urban Type 4:PCA2	-0.16±0.1
			Urban Type 5:PCA2	-0.18±0.1
			Urban Type 6:PCA2	-0.1±0.11
			Urban Type 8:PCA2	-0.19±0.1
			Urban Type 9:PCA2	-0.13±0.1
Month:PCA1	$0.23_{(1,133)}$	0.6348	Month:PCA1	0.01±0.01
Month:PCA2	$0.05_{(1,133)}$	0.8303	Month:PCA2	0.01±0.01

Table S8.

Full model output from repeated measures linear model of insect species richness for the high insect diversity season (March - May). Significant effects are bolded.

Fixed Effects	F	p-value	Model Parameters	coefficients (±SE)
**(Intercept)**	**$273.29_{(1,43)}$**	**<0.0001**	(Intercept)	-2.12±11.66
PCA1	$1.01_{(1,43)}$	0.3205	PCA1	-0.43±2.35
PCA2	$2.07_{(1,43)}$	0.1576	PCA2	-9.2±8.92
Month	$0.16_{(1,43)}$	0.6908	Month	1.37±1.16
Compost	$0.41_{(1,17)}$	0.5297	Compost Present	-0.82±0.64
Mulch	$1.4_{(1,17)}$	0.2537	Mulch Present	1.98±0.88
**Drought Tolerant**	**$9.09_{(1,17)}$**	**0.0078**	Drought Tolerant Present	-0.09±1.51
Native	$0.24_{(1,17)}$	0.6303	Native Present	0.66±1.5
Lawn	$1.23_{(1,17)}$	0.2833	Lawn Present	-0.46±0.71
Water	$3.05_{(1,17)}$	0.0987	Water Frequent	-1.95±0.86
Urban Type	$1.07_{(6,17)}$	0.4169	Urban Type 3	1.08±10.07
			Urban Type 4	-0.4±10.41
			Urban Type 5	0.54±10.27
			Urban Type 6	2.94±11.35
			Urban Type 8	3.27±10.24
			Urban Type 9	3.32±10.21
Urban Type:PCA1	$0.19_{(6,43)}$	0.9786	Urban Type 3:PCA1	-0.18±1.32
			Urban Type 4:PCA1	-0.21±1.4
			Urban Type 5:PCA1	-0.13±1.36
			Urban Type 6:PCA1	0.21±1.4
			Urban Type 8:PCA1	0.43±1.36
			Urban Type 9:PCA1	0.14±1.35
Urban Type:PCA2	$0.51_{(6,43)}$	0.7942	Urban Type 3:PCA2	1.53±7.46
			Urban Type 4:PCA2	-0.51±7.81
			Urban Type 5:PCA2	3.47±7.62
			Urban Type 6:PCA2	1.42±8.26
			Urban Type 8:PCA2	4.64±7.64
			Urban Type 9:PCA2	4.14±7.64
Month:PCA1	$3.72_{(1,43)}$	0.0603	Month:PCA1	0.26±0.41
Month:PCA2	$4.1_{(1,43)}$	0.0592	Month:PCA2	1.85±0.92

Table S9.

Full model output from repeated measures linear model of insect abundance for the high insect diversity season (March - May). Significant effects are bolded.

Fixed Effects	F	p-value	Model Parameters	coefficients (±SE)
**(Intercept)**	**$263.58_{(1,43)}$**	**<0.0001**	(Intercept)	9.83±14.35
PCA1	$2.81_{(1,43)}$	0.1012	PCA1	0.4±2.9
**PCA2**	**$5.44_{(1,43)}$**	**0.0244**	PCA2	0.01±11.07
Month	$1.25_{(1,43)}$	0.2693	Month	0.88±1.5
Compost	$2.84_{(1,17)}$	0.1103	Compost Present	-1.34±0.86
Mulch	$0.49_{(1,17)}$	0.4955	Mulch Present	1.75±1.19
**Drought Tolerant**	**$6.91_{(1,17)}$**	**0.0176**	Drought Tolerant Present	2.38±2.04
Native	$0.54_{(1,17)}$	0.4713	Native Present	-1.74±2.03
Lawn	$0.58_{(1,17)}$	0.4559	Lawn Present	-0.46±0.96
Water	$2.26_{(1,17)}$	0.1512	Water Frequent	-2.07±1.16
Urban Type	$0.86_{(6,17)}$	0.5418	Urban Type 3	-5.43±12.17
			Urban Type 4	-6.9±12.61
			Urban Type 5	-8.47±12.43
			Urban Type 6	-4.22±13.66
			Urban Type 8	-5.07±12.39
			Urban Type 9	-5.15±12.36
Urban Type:PCA1	$0.19_{(6,43)}$	0.9778	Urban Type 3:PCA1	-0.39±1.52
			Urban Type 4:PCA1	-0.57±1.62
			Urban Type 5:PCA1	-0.95±1.57
			Urban Type 6:PCA1	-0.24±1.61
			Urban Type 8:PCA1	0.19±1.57
			Urban Type 9:PCA1	-0.69±1.57
Urban Type:PCA2	$0.98_{(6,43)}$	0.4528	Urban Type 3:PCA2	-3.82±9.13
			Urban Type 4:PCA2	-5.04±9.55
			Urban Type 5:PCA2	-4.69±9.32
			Urban Type 6:PCA2	-5.19±10.04
			Urban Type 8:PCA2	-2.05±9.35
			Urban Type 9:PCA2	-3.41±9.37
Month:PCA1	$1.1_{(1,43)}$	0.3005	Month:PCA1	0.23±0.53
Month:PCA2	$1.38_{(1,43)}$	0.2461	Month:PCA2	1.38±1.18

Table S10.

Full model output from repeated measures linear model of insect species richness for the intermediate insect diversity season (June - August). Significant effects are bolded.

Fixed Effects	F	p-value	Model Parameters	coefficients (±SE)
**(Intercept)**	**194.82$_{(1,43)}$**	**<0.0001**	(Intercept)	19.2±5.24
PCA1	1.91$_{(1,43)}$	0.1736	PCA1	2.59±2.26
**PCA2**	**5.7$_{(1,43)}$**	**0.0214**	PCA2	-5.7±2.55
**Month**	**6.26$_{(1,43)}$**	**0.0163**	Month	-2.35±0.86
Compost	0.14$_{(1,17)}$	0.7091	Compost Present	-0.32±0.61
Mulch	3.36$_{(1,17)}$	0.0843	Mulch Present	1.38±0.88
**Drought Tolerant**	**7.27$_{(1,17)}$**	**0.0153**	Drought Tolerant Present	2.36±1.52
Native	0.69$_{(1,17)}$	0.4174	Native Present	-1.29±1.52
Lawn	0.02$_{(1,17)}$	0.898	Lawn Present	0.31±0.7
Water	0.98$_{(1,17)}$	0.3359	Water Frequent	-0.81±0.82
**Urban Type**	**3.15$_{(6,17)}$**	**0.029**	Urban Type 3	-0.07±2.95
			Urban Type 4	0.89±3.22
			Urban Type 5	-5.76±3.05
			Urban Type 6	-1.14±3.11
			Urban Type 8	-3.74±3.62
			Urban Type 9	3.61±3.66
Urban Type:PCA1	0.57$_{(6,43)}$	0.7501	Urban Type 3:PCA1	-0.54±0.89
			Urban Type 4:PCA1	-0.5±0.92
			Urban Type 5:PCA1	-0.79±0.92
			Urban Type 6:PCA1	0.29±1.04
			Urban Type 8:PCA1	-0.13±0.92
			Urban Type 9:PCA1	-0.46±0.91
Urban Type:PCA2	1.16$_{(6,43)}$	0.3433	Urban Type 3:PCA2	-1.27±1.93
			Urban Type 4:PCA2	-1.56±1.94
			Urban Type 5:PCA2	0.72±2.01
			Urban Type 6:PCA2	0.56±2.15
			Urban Type 8:PCA2	0.02±2.14
			Urban Type 9:PCA2	-3.06±2.09
**Month:PCA1**	**6.79$_{(1,43)}$**	**0.0125**	Month:PCA1	-0.23±0.31
**Month:PCA2**	**7.27$_{(1,43)}$**	**0.01**	Month:PCA2	1.01±0.37

Table S11.

Full model output from repeated measures linear model of insect abundance for the intermediate insect diversity season (June - August). Significant effects are bolded.

Fixed Effects	F	p-value	Model Parameters	coefficients (±SE)
**(Intercept)**	**$321.55_{(1,43)}$**	**<0.0001**	(Intercept)	2.21±1.25
PCA1	$1.82_{(1,43)}$	0.1838	PCA1	0.5±0.53
**PCA2**	**$9.45_{(1,43)}$**	**0.0037**	PCA2	-0.4±0.6
**Month**	**$3.93_{(1,43)}$**	**0.0538**	Month	-0.12±0.2
Compost	$0.002_{(1,17)}$	0.9599	Compost Present	-0.19±0.17
**Mulch**	**$5.97_{(1,17)}$**	**0.0257**	Mulch Present	0.54±0.24
**Drought Tolerant**	**$4.27_{(1,17)}$**	**0.0544**	Drought Tolerant Present	0.22±0.42
Native	$0.02_{(1,17)}$	0.904	Native Present	0.04±0.42
Lawn	$0.01_{(1,17)}$	0.9388	Lawn Present	0.08±0.19
Water	$0.48_{(1,17)}$	0.4969	Water Frequent	-0.14±0.23
Urban Type	$2.48_{(6,17)}$	0.0659	Urban Type 3	0.41±0.72
			Urban Type 4	0.03±0.79
			Urban Type 5	-0.97±0.75
			Urban Type 6	0.12±0.77
			Urban Type 8	-0.68±0.88
			Urban Type 9	0.38±0.89
Urban Type:PCA1	$0.13_{(6,43)}$	0.9916	Urban Type 3:PCA1	0.04±0.2
			Urban Type 4:PCA1	0.04±0.21
			Urban Type 5:PCA1	0.03±0.21
			Urban Type 6:PCA1	-0.01±0.24
			Urban Type 8:PCA1	-0.03±0.21
			Urban Type 9:PCA1	-0.03±0.21
Urban Type:PCA2	$0.89_{(6,43)}$	0.5116	Urban Type 3:PCA2	-0.31±0.45
			Urban Type 4:PCA2	-0.15±0.45
			Urban Type 5:PCA2	-0.05±0.47
			Urban Type 6:PCA2	0.21±0.5
			Urban Type 8:PCA2	0.18±0.5
			Urban Type 9:PCA2	-0.26±0.49
Month:PCA1	$1.82_{(1,43)}$	0.1843	Month:PCA1	-0.06±0.07
Month:PCA2	$0.28_{(1,43)}$	0.5966	Month:PCA2	0.05±0.09

Table S12.

The list of indicator taxa of each season from collections made throughout the year. Included in the table are the season, the indicator taxa, the A indice (mean abundance of the species within a season in this case divided by the sum of the mean abundance in all seasons) and B indice (the frequency with which a species occurs within a season) used to calculate the indicator value (IndVal; Dufrêne and Legendre 1997, de Cáceres and Legendre 2009), and a p-value (P).

**Season**	**Indicator Species**	**A**	**B**	**IndVal**	**P**
Sept - Feb	*Drosophila gentica*	0.73	0.16	0.34	0.0285
Sept - Feb	*Megaselia arizonensis*	0.56	0.23	0.36	0.0132
Sept - Feb	*Megaselia hirticaudata*	0.72	0.10	0.27	0.0140
Sept - Feb	*Megaselia wongae*	0.76	0.09	0.27	0.0148
March - May	*Allograpta obliqua*	0.65	0.42	0.52	0.0001
March - May	*Anevrina* sp1	0.75	0.23	0.42	0.0001
March - May	*Coboldia fuscipes*	0.61	0.13	0.29	0.0169
March - May	*Conicera similis*	0.67	0.56	0.61	0.0001
March - May	*Conicera tibialis*	0.83	0.37	0.55	0.0001
March - May	*Drosophila busckii*	0.76	0.34	0.51	0.0001
March - May	*Drosophila immigrans*	0.69	0.44	0.55	0.0001
March - May	*Drosophila occidentalis*	0.55	0.32	0.42	0.0012
March - May	*Drosophila simulans*	0.48	0.32	0.39	0.0151
March - May	*Eupeodes volucris*	0.67	0.18	0.35	0.0006
March - May	*Gitona americana*	0.73	0.14	0.32	0.0490
March - May	*Megaselia agarici*	0.50	0.99	0.71	0.0001
March - May	*Megaselia albicaudata*	0.46	0.42	0.44	0.0234
March - May	*Megaselia armstrongorum*	0.84	0.89	0.87	0.0001
March - May	*Megaselia atrox*	0.62	0.27	0.41	0.0020
March - May	*Megaselia basispinata*	0.67	0.39	0.51	0.0001
March - May	*Megaselia creasoni*	0.76	0.51	0.63	0.0001
March - May	*Megaselia fujiokai*	0.70	0.47	0.57	0.0001
March - May	*Megaselia halterata*	0.67	0.84	0.75	0.0001
March - May	*Megaselia largifrontalis*	0.73	0.53	0.62	0.0001
March - May	*Megaselia lombardorum*	0.61	0.92	0.75	0.0001
March - May	*Megaselia mikejohnsoni*	0.78	0.47	0.60	0.0001
March - May	*Megaselia nigra*	0.66	0.88	0.76	0.0001
March - May	*Megaselia pleuralis*	0.71	0.57	0.64	0.0001
March - May	*Megaselia rodriguezorum*	0.77	0.14	0.33	0.0003

March - May	*Megaselia rufipes*	0.79	0.40	0.56	0.0001
March - May	*Megaselia sulphurizona*	0.49	0.93	0.68	0.0002
March - May	*Quateiella quatei*	0.46	0.41	0.44	0.0315
March - May	*Repleta* sp1	0.57	0.27	0.39	0.0070
March - May	*Swammerdamella marginata*	0.70	0.63	0.67	0.0001
March - May	*Trophodeinus furcatus*	0.63	0.17	0.32	0.0253
June - Aug	*Agraulis vanilla*	0.77	0.16	0.35	0.0003
June - Aug	*Apis mellifera*	0.60	0.64	0.62	0.0020
June - Aug	*Autographa californica*	0.66	0.22	0.38	0.0002
June - Aug	*Beckerina* sp1	0.93	0.49	0.67	0.0001
June - Aug	*Brephidium exile*	0.84	0.16	0.36	0.0001
June - Aug	*Dioprosopa clavata*	0.66	0.63	0.65	0.0001
June - Aug	*Halictus tripartitus*	0.73	0.42	0.55	0.0001
June - Aug	*Lasioglossum* sp1	0.62	0.77	0.69	0.0001
June - Aug	*Megaselia berndseni*	0.60	0.50	0.55	0.0001
June - Aug	*Megaselia francoae*	0.41	0.44	0.43	0.0046
June - Aug	*Megaselia hansonix*	0.44	0.38	0.41	0.0244
June - Aug	*Megaselia kelleri*	0.58	0.30	0.42	0.0003
June - Aug	*Megaselia oxboroughae*	0.69	0.50	0.59	0.0001
June - Aug	*Megaselia pisanoi*	0.84	0.13	0.33	0.0024
June - Aug	*Megaselia sidneyae*	0.56	0.59	0.57	0.0001
June - Aug	*Megaselia steptoeae*	0.59	0.49	0.54	0.0074
June - Aug	*Megaselia wiegmanae*	0.48	0.72	0.59	0.0011
June - Aug	*Megachile* sp1	0.61	0.18	0.33	0.0006
June - Aug	*Metopina* sp1	0.47	0.24	0.34	0.0378
June - Aug	*Nephrotoma wulpiana*	0.53	0.27	0.38	0.0015
June - Aug	*Paragus haemorrhous*	0.75	0.60	0.67	0.0001
June - Aug	*Phalacrotophora halictorum*	0.65	0.36	0.48	0.0001

Table S13.

Results from pairwise PERMANOVAs comparing insect species composition among urban types during September – February collections (A), March – May collections (B), and June – August collectons (C). Include in each table is a column representing the pair of compared urban types, a t-value from the pairwise test, a permutations-based p-value, and the number of unique permutations produce from 9999 possible runs. Urban types with significantly different insect species compositions are in bold.

A)

**Urban Type Pairs**	**t**	**p-value**	**Unique Perms**
9,8	1.09	0.1995	9877
9,6	0.86	0.8933	9883
9,5	1.03	0.3501	9873
9,4	1.06	0.2589	9884
**9,3**	**1.31**	**0.0065**	**9865**
9,1	1.10	0.1991	9882
8,6	1.10	0.2012	9875
8,5	1.09	0.1993	9873
8,4	1.12	0.1415	9854
**8,3**	**1.22**	**0.032**	**9879**
8,1	1.16	0.1431	9890
6,5	1.16	0.1345	9896
6,4	1.16	0.1369	9905
**6,3**	**1.20**	**0.0546**	**9874**
6,1	1.14	0.3453	9949
5,4	0.88	0.8702	9881
5,3	1.11	0.1409	9868
5,1	1.18	0.1223	9900
4,3	1.16	0.0729	9857
4,1	1.12	0.2051	9904
3,1	1.09	0.2035	9869

B)

Urban Type Pairs	t	p-value	Unique Perms
9,8	0.94	0.6659	9894
9,6	0.92	0.699	9900
9,5	0.94	0.6472	9900
9,4	1.12	0.1819	9910
9,3	1.09	0.2276	9903
9,1	1.01	0.4476	9907
8,6	1.04	0.3798	9907
8,5	0.98	0.5082	9899
**8,4**	**1.46**	**0.0147**	**9912**
8,3	1.22	0.0621	9872
8,1	1.20	0.1771	9917
6,5	1.01	0.4413	9920
6,4	1.34	0.0833	9913
6,3	1.04	0.3581	9911
6,1	1.37	0.2618	4115
5,4	1.11	0.2365	9900
5,3	1.11	0.2057	9896
5,1	1.12	0.312	9932
**4,3**	**1.27**	**0.0404**	**9898**
4,1	1.11	0.2895	9938
3,1	1.04	0.3591	9899

C)

Urban Type Pairs	t	p-value	Unique Perms
9,8	0.82	0.9414	9886
9,6	1.27	0.0961	9911
9,5	1.08	0.2809	9910
9,4	1.05	0.3478	9893
**9,3**	**1.30**	**0.0301**	**9898**
9,1	1.12	0.2278	9900
8,6	1.37	0.0803	9934
8,5	1.10	0.279	9923
8,4	1.17	0.1502	9895
8,3	1.26	0.0599	9916
8,1	1.24	0.167	9932
6,5	1.53	0.0797	9938
**6,4**	**1.51**	**0.0503**	**9927**
**6,3**	**1.30**	**0.0511**	**9897**
6,1	1.68	0.1089	4117
5,4	1.29	0.1019	9920
5,3	1.56	0.0141	9908
5,1	1.23	0.2462	9926
4,3	1.11	0.1869	9878
4,1	1.27	0.1484	9939
3,1	1.04	0.353	9882

Table S14.

The list of indicator taxa of significantly different urban types (A), and sites with and without drought tolerant plants (B), native plants (C), and compost (D) from collection made from September – February. Included in each table are the indicator taxa, the A and B indices used to calculate the indicator value (IndVal), and a p-value (P).

A)

**Urban Type**	**Indicator Species**	**A**	**B**	**IndVal**	**P**
3	*Repleta* sp1	0.52	0.38	0.45	0.0200
6	*Megaselia agarici*	0.32	1.00	0.57	0.0166
6	*Megaselia berndseni*	0.40	0.42	0.41	0.0500
6	*Megaselia wiegmanae*	0.45	0.83	0.61	0.0028
6	*Megaselia wongae*	0.55	0.42	0.48	0.0081
6	*Nephrotoma suturalis*	0.59	0.33	0.44	0.0111
6	*Swammerdamella marginata*	0.63	0.58	0.61	0.0029
8	*Chonocephalus bentacaisei*	0.59	0.50	0.54	0.0210
8	*Megaselia donahuei*	0.46	0.43	0.45	0.0251
8	*Megaselia marquezi*	0.57	0.57	0.57	0.0039
8	*Megaselia scalaris*	0.57	0.33	0.43	0.0272
9	*Megaselia sacatelensis*	0.53	0.43	0.48	0.0253

B)

**Drought Tolerant**	**Indicator Species**	**A**	**B**	**IndVal**	**P**
Yes	*Allograpta exotica*	1.00	0.05	0.22	0.0337
Yes	*Allograpta obliqua*	0.81	0.17	0.37	0.0034
Yes	*Apis mellifera*	0.66	0.57	0.61	0.0066
Yes	*Ceratina* sp1	0.82	0.17	0.37	0.0084
Yes	*Dioprosopa clavata*	0.74	0.50	0.61	0.0008
Yes	*Gitona bivisualis*	0.91	0.17	0.39	0.0005
Yes	*Lasioglossum* sp1	0.61	0.58	0.60	0.0267
Yes	*Megaselia albicaudata*	0.78	0.38	0.55	0.001
Yes	*Megaselia barberi*	0.97	0.30	0.54	0.0012
Yes	*Megaselia basispinata*	0.84	0.18	0.39	0.0064
Yes	*Megaselia fujiokai*	0.88	0.23	0.45	0.0026
Yes	*Megaselia halterata*	0.59	0.53	0.56	0.0329
Yes	*Megaselia heini*	0.80	0.35	0.53	0.0017
Yes	*Megaselia hentschkeae*	0.82	0.12	0.31	0.0133
Yes	*Megaselia hoggorum*	0.98	0.13	0.36	0.0006
Yes	*Megaselia largifrontalis*	0.66	0.50	0.57	0.0372
Yes	*Megaselia lombardorum*	0.69	0.87	0.77	0.0001
Yes	*Megaselia mikejohnsoni*	0.83	0.30	0.50	0.0152
Yes	*Megaselia pisanoi*	1.00	0.08	0.29	0.0024
Yes	*Megaselia rufipes*	0.85	0.15	0.36	0.0176
Yes	*Megaselia sidneyae*	0.91	0.42	0.62	0.0001
Yes	*Megaselia steptoeae*	0.68	0.55	0.61	0.0358
Yes	*Megaselia sulphurizona*	0.65	0.93	0.78	0.0001
Yes	*Metopina* sp1	0.81	0.20	0.40	0.0278
Yes	*Quateiella quatei*	0.73	0.38	0.53	0.0024
Yes	*Sphecodes* sp1	0.64	0.43	0.53	0.0088
Yes	*Toxomerus marginatus*	0.74	0.32	0.48	0.0018

C)

**Native**	**Indicator Species**	**A**	**B**	**IndVal**	**P**
Yes	*Allograpta exotica*	1.00	0.06	0.24	0.02
Yes	*Allograpta obliqua*	0.83	0.19	0.39	0.0009
Yes	*Apis mellifera*	0.65	0.56	0.60	0.02
Yes	*Beckerina* sp1	0.76	0.10	0.28	0.04
Yes	*Ceratina* sp1	0.83	0.21	0.42	0.001
Yes	*Dioprosopa clavata*	0.62	0.48	0.55	0.04
Yes	*Gitona americana*	0.87	0.15	0.36	0.006
Yes	*Lasioglossum* sp1	0.62	0.57	0.60	0.03
Yes	*Megaselia albicaudata*	0.65	0.33	0.47	0.05
Yes	*Megaselia barberi*	0.86	0.33	0.53	0.001
Yes	*Megaselia basispinata*	0.84	0.19	0.40	0.007
Yes	*Megaselia fujiokai*	0.76	0.23	0.42	0.02
Yes	*Megaselia hentschkeae*	0.84	0.13	0.33	0.007
Yes	*Megaselia lombardorum*	0.59	0.85	0.71	0.002
Yes	*Megaselia mikejohnsoni*	0.84	0.30	0.50	0.02
Yes	*Megaselia pisanoi*	0.94	0.08	0.28	0.01
Yes	*Megaselia rufipes*	0.85	0.15	0.36	0.03
Yes	*Megaselia sidneyae*	0.67	0.38	0.50	0.01
Yes	*Megaselia sulphurizona*	0.56	0.92	0.71	0.01
Yes	*Metopina* sp1	0.82	0.20	0.41	0.03
Yes	*Sphecodes* sp1	0.65	0.43	0.53	0.01
Yes	*Toxomerus marginatus*	0.68	0.29	0.45	0.01

D)

Compost	Indicator Species	A	B	IndVal	P
No	*Megaselia agarici*	0.69	0.91	0.79	0.01
No	*Megaselia carthayensis*	0.83	0.30	0.50	0.03
No	*Megaselia largifrontalis*	0.71	0.46	0.57	0.05
No	*Megaselia nigra*	0.74	0.56	0.64	0.02
No	*Megaselia sacatelensis*	0.84	0.24	0.45	0.03
No	*Megaselia tecticauda*	0.97	0.14	0.37	0.01
No	*Puliciphora occidentalis*	0.90	0.33	0.54	0.004
Yes	*Chonocephalus bentacaisei*	0.78	0.42	0.57	0.0003
Yes	*Drosophila busckii*	0.74	0.13	0.31	0.03
Yes	*Drosophila immigrans*	0.75	0.17	0.35	0.04
Yes	*Drosophila simulans*	0.77	0.13	0.32	0.05
Yes	*Megaselia hansonix*	0.64	0.37	0.49	0.02
Yes	*Paragus haemorrhous*	0.69	0.32	0.47	0.01

Table S15.

The list of indicator taxa of each urban types (A), and sites with and without drought tolerant plants (B), or with and without compost (C) from collection made from March – May. Included in each table are the indicator taxa, the A and B indices used to calculate the indicator value (IndVal), and a p-value (P).

A)

**Urban Type**	**Indicator Species**	**A**	**B**	**IndVal**	**P**
3	*Gitona americana*	0.85	0.38	0.57	0.0315
4	*Trophodeinus furcatus*	0.89	0.83	0.86	0.0003
8	*Megaselia rufipes*	0.52	0.73	0.62	0.0273

B)

Drought Tolerant	Indicator Species	A	B	IndVal	P
Yes	*Allograpta exotica*	0.76	0.23	0.42	0.0301
Yes	*Apis mellifera*	0.64	0.80	0.72	0.0168
Yes	*Dioprosopa clavata*	0.88	0.37	0.57	0.0005
Yes	*Eupeodes volucris*	0.79	0.30	0.49	0.0135
Yes	*Gitona americana*	0.96	0.30	0.54	0.0020
Yes	*Halictus tripartitus*	0.71	0.27	0.44	0.0343
Yes	*Hylaeus* sp1	0.87	0.20	0.42	0.0086
Yes	*Lasioglossum* sp1	0.71	0.63	0.67	0.0130
Yes	*Megaselia arizonensis*	0.75	0.37	0.52	0.0107
Yes	*Megaselia barberi*	0.87	0.63	0.74	0.0021
Yes	*Megaselia basispinata*	0.75	0.53	0.63	0.0100
Yes	*Megaselia berndseni*	0.63	0.60	0.62	0.0218
Yes	*Megaselia donahuei*	0.74	0.33	0.50	0.0286
Yes	*Megaselia francoae*	0.74	0.40	0.55	0.0288
Yes	*Megaselia fujiokai*	0.69	0.63	0.66	0.0197
Yes	*Megaselia hardingorum*	0.92	0.13	0.35	0.0336
Yes	*Megaselia heini*	0.88	0.43	0.62	0.0005
Yes	*Megaselia hoggorum*	0.97	0.20	0.44	0.0067
Yes	*Megaselia kelleri*	0.77	0.23	0.42	0.0276
Yes	*Megaselia mikejohnsoni*	0.91	0.63	0.76	0.0028
Yes	*Megaselia rufipes*	0.74	0.53	0.63	0.0212
Yes	*Megaselia sidneyae*	0.82	0.40	0.57	0.0319
Yes	*Megaselia sulphurizona*	0.65	0.97	0.79	0.0277

C)

Compost	Indicator Species	A	B	IndVal	P
No	*Megaselia agarici*	0.76	1.00	0.87	0.0001
No	*Megaselia albicaudata*	0.75	0.48	0.60	0.0482
No	*Megaselia armstrongorum*	0.78	0.92	0.85	0.0005
No	*Megaselia atrox*	0.86	0.35	0.55	0.0208
No	*Megaselia lombardorum*	0.67	0.93	0.79	0.0043
No	*Megaselia nigra*	0.68	0.92	0.79	0.0144
No	*Megaselia oxboroughae*	0.93	0.30	0.53	0.0226
No	*Megaselia sacatelensis*	0.79	0.33	0.51	0.0444
No	*Puliciphora occidentalis*	0.73	0.43	0.56	0.0249
Yes	*Dioprosopa.clavata*	0.75	0.30	0.48	0.0426
Yes	*Megaselia marquezi*	0.73	0.83	0.78	0.0260
Yes	*Megaselia ruficornis*	0.83	0.17	0.37	0.0369
Yes	*Megaselia steptoeae*	0.80	0.37	0.54	0.0100
Yes	*Paragus.haemorrhous*	0.70	0.43	0.55	0.0445
Yes	*Spiniphora bergenstammi*	0.84	0.23	0.44	0.0222

Table S16.

The list of indicator taxa of each urban type (A) and sites with and without drought tolerant plants (B), or native plants (C) from collection made from June – August. Included in each table are the indicator taxa, the A and B indices used to calculate the indicator value (IndVal), and a p-value (P).

A)

**Urban Type**	**Indicator Species**	**A**	**B**	**IndVal**	**P**
3	*Phalacrotophora halictorum*	0.42	0.86	0.60	0.0192
4	*Megaselia albicaudata*	0.47	0.83	0.63	0.0135
4	*Trophodeinus furcatus*	0.58	0.67	0.62	0.0104
6	*Conicera similis*	0.43	0.83	0.60	0.024
6	*Megaselia berndseni*	0.44	1.00	0.66	0.0266
6	*Megaselia carthayensis*	0.52	0.83	0.66	0.0146
6	*Megaselia oxboroughae*	0.76	1.00	0.87	0.0001
6	*Megaselia wiegmanae*	0.52	1.00	0.72	0.0002
6	*Puliciphora occidentalis*	0.55	0.83	0.68	0.0073
6	*Toxomerus marginatus*	0.72	0.67	0.69	0.0032

B)

Drought Tolerant	Indicator Species	A	B	IndVal	P
Yes	*Allograpta exotica*	0.86	0.17	0.38	0.0330
Yes	*Beckerina* sp1	0.65	0.63	0.64	0.0337
Yes	*Chonocephalus bentacaisei*	0.67	0.53	0.60	0.0375
Yes	*Coboldia fuscipes*	1.00	0.10	0.32	0.0333
Yes	*Dioprosopa clavata*	0.77	0.87	0.82	0.0001
Yes	*Drosophila simulans*	0.84	0.27	0.47	0.0040
Yes	*Gitona americana*	0.93	0.27	0.50	0.0013
Yes	*Lasioglossum* sp1	0.69	0.90	0.79	0.0034
Yes	*Megaselia armstrongorum*	0.86	0.53	0.68	0.0009
Yes	*Megaselia donahuei*	0.79	0.27	0.46	0.0278
Yes	*Megaselia fujiokai*	0.78	0.37	0.53	0.0057
Yes	*Megaselia hoggorum*	0.99	0.20	0.45	0.0042
Yes	*Megaselia pisanoi*	0.99	0.27	0.51	0.0027
Yes	*Megaselia sidneyae*	0.74	0.73	0.74	0.0049
Yes	*Megaselia sulphurizona*	0.67	0.93	0.79	0.0080
Yes	*Megaselia wongae*	0.95	0.13	0.36	0.0098
Yes	*Metopina* sp1	0.87	0.50	0.66	0.0002
Yes	*Paragus haemorrhous*	0.68	0.70	0.69	0.0469
Yes	*Quateiella quatei*	0.84	0.50	0.65	0.0033
Yes	*Repleta* sp1	0.74	0.33	0.50	0.0183
Yes	*Swammerdamella marginata*	0.72	0.67	0.69	0.0041

C)

Native	Indicator Species	A	B	IndVal	P
Yes	*Allograpta obliqua*	0.88	0.19	0.40	0.0201
Yes	*Anevrina* sp1	0.93	0.11	0.32	0.0419
Yes	*Beckerina* sp1	0.66	0.63	0.64	0.0363
Yes	*Chonocephalus bentacaisei*	0.69	0.52	0.60	0.0453
Yes	*Dioprosopa clavata*	0.79	0.89	0.84	0.0001
Yes	*Drosophila simulans*	0.86	0.30	0.50	0.0011
Yes	*Gitona americana*	0.86	0.22	0.44	0.0232
Yes	*Lasioglossum* sp1	0.66	0.89	0.77	0.013
Yes	*Megaselia armstrongorum*	0.87	0.56	0.69	0.0003
Yes	*Megaselia creasoni*	0.74	0.22	0.41	0.0179
Yes	*Megaselia fujiokai*	0.78	0.37	0.54	0.0061
Yes	*Megaselia hansonix*	0.66	0.52	0.59	0.0423
Yes	*Megaselia pisanoi*	0.97	0.19	0.43	0.0486
Yes	*Megaselia wongae*	0.95	0.15	0.38	0.0061
Yes	*Megachile* sp1	0.69	0.30	0.45	0.0498
Yes	*Metopina* sp1	0.86	0.52	0.67	0.0003
Yes	*Paragus haemorrhous*	0.70	0.70	0.70	0.0296

Table S17.

Average distances (±SE) of dispersal from the centroid for each urban type during June through August collections. Included are the urban type and the number of collections within each urban type. Mean distances do not differ among urban types that share a superscript letter.

**Urban Type**	**Collections**	**Distance**
1	3	24.2±2.0^{a}
3	21	42.1±1.4^{c}
4	12	36.6±1.6^{b}
5	12	43.2±1.8^{c}
6	6	25.9±1.9^{a}
8	15	40.9±2.3^{b,c}
9	21	43.3±2.0^{b,c}

SI Figure Legends

Figure S1. Average daily precipitation (mm; black bars), maximum air temperature (C, dark grey line), and minimum air temperature (C, light grey line) per month for the urban center of Los Angeles County collected from the University of Southern California from 1981 – 2010. Data were obtained via the Western Regional Climate Center in association with the National Weather Service.

Figure S2. Locations of the 30 collection sites from across Los Angeles County. Sites are color coded by urban type.

Figure S3. Species accumulation curves for A) Bees, B) Lepidoptera, C) Drosophilidae, D) Phoridae, E) Scatopsidae, F) Syrphidae, and G) Tipulidae based on the 360 collections. The darker line in each graph represents the total number of species collected after each collection. The thinner lines represent the upper and lower 95%CI (S_{est}) for the number of species predicted for each group. In total, we collected 25 species of bees, 24 species of Lepidoptera, 22 species of Drosophilidae, 99 species of Phoridae, 7 species of Scatopsidae, 29 species of Syrphidae, and 11 species of Tipulidae.

Figure S4. Insect species richness/trap day over daily maximum air temperature values measured at all sites during August.

Figure S5**.** Insect species richness/trap day over daily maximum percent relative humidity (A) and daily minimum percent relative humidity (B) values measured at all sites during June.

Figure S6. NMDS ordinations of species composition during September through February (stress =0.15). Axes were chosen for each plot to best visualize differences among urban types (A), and presence or absence of drought-tolerant plants (B), native plants (C), or compost (D). Each point represents an individual collections. A legend is provided in the top right of each plot. Ellipses indicate the standard error measurements around the centroid of each fixed effect group in an ordination. Vectors indicate significant correlations between species composition and a measured environmental variable. The length of each vector is proportional to the strength of the correlation.

Figure S7. NMDS ordinations of species composition during June through August (stress =0.13). Axes were chosen for each plot to best visualize differences among urban types (A), the presence of drought-tolerant plants (B), and the presence of native plants (C). Each point represents an individual collections. A legend is provided in the top right of each plot. Ellipses indicate the standard error measurements around the centroid of each fixed effect group in an ordination.

Figure S8. NMDS ordinations of species composition during March through May (stress =0.13). Axes were chosen for each plot to best visualize differences among urban types (A), the presence of drought-tolerant plants (B), and the presence of compost (C). Each point represents an individual collections. A legend is provided in the top right of each plot. Ellipses indicate the standard error measurements around the centroid of each fixed effect group in an ordination.

SI Figures

Figure S1.

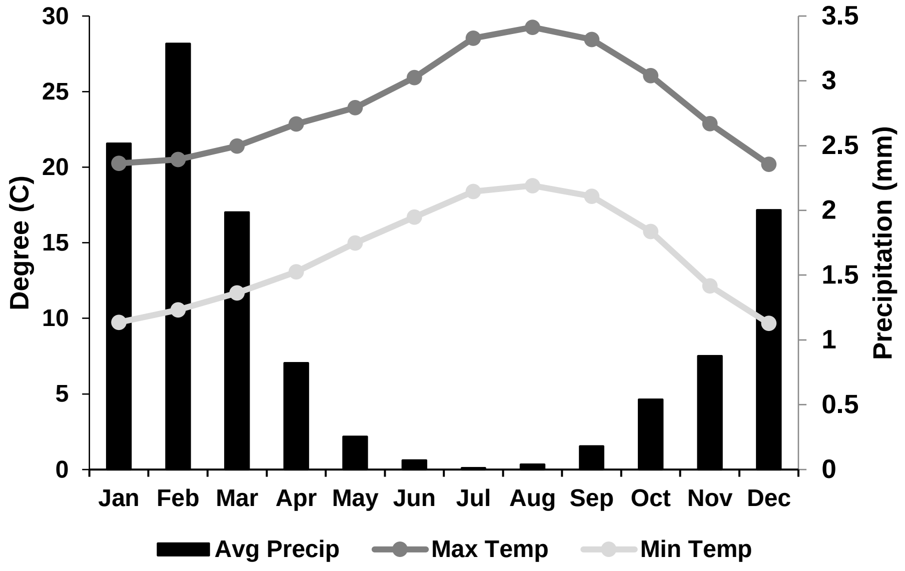

Figure S2.

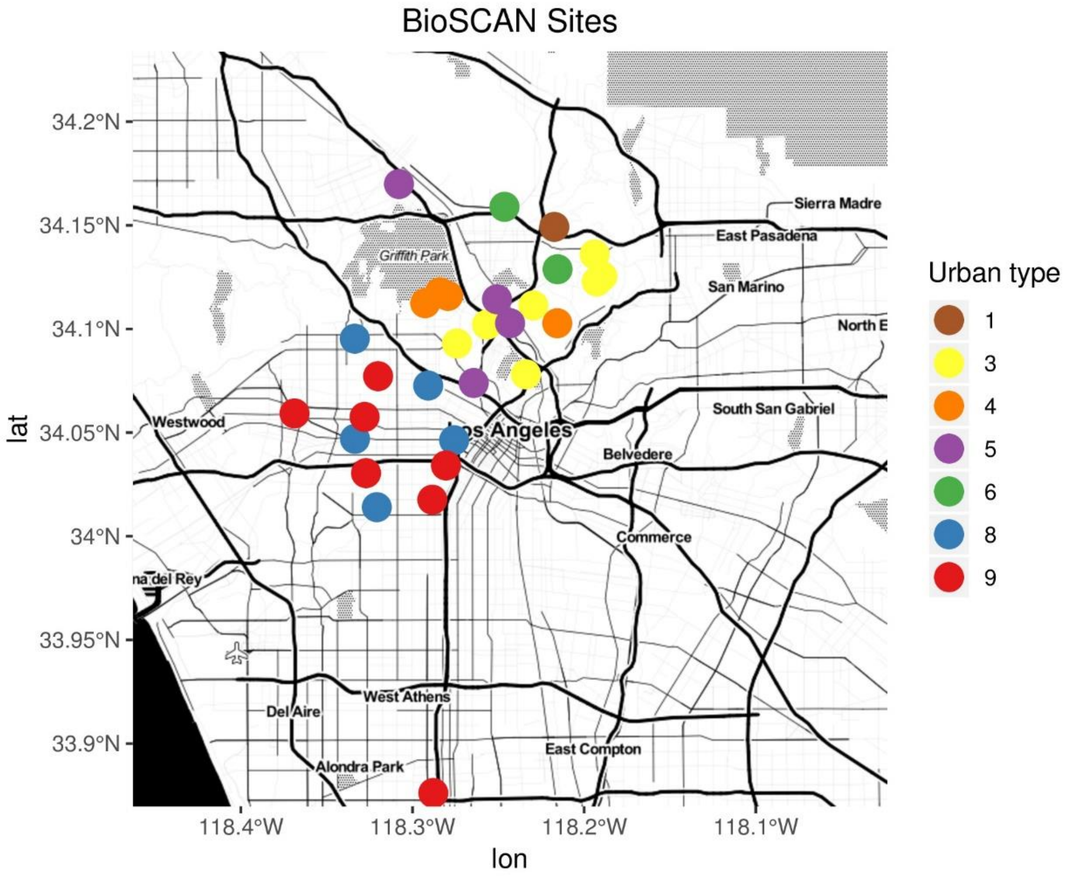
BioSCAN Sites
34.2°N
34.15°N
34.1°N
34.05°N
34°N
33.95°N
33.9°N
lat
118.4°W
118.3°W
118.2°W
118.1°W
lon
Sierra Madre
East Pasadena
Griffith Park
San Marino
North E
South San Gabriel
Westwood
Los Angeles
Belvedere
Commerce
na del Rey
West Athens
Del Aire
East Compton
Alondra Park
Urban type
1
3
4
5
6
8
9

Figure S3.

A)

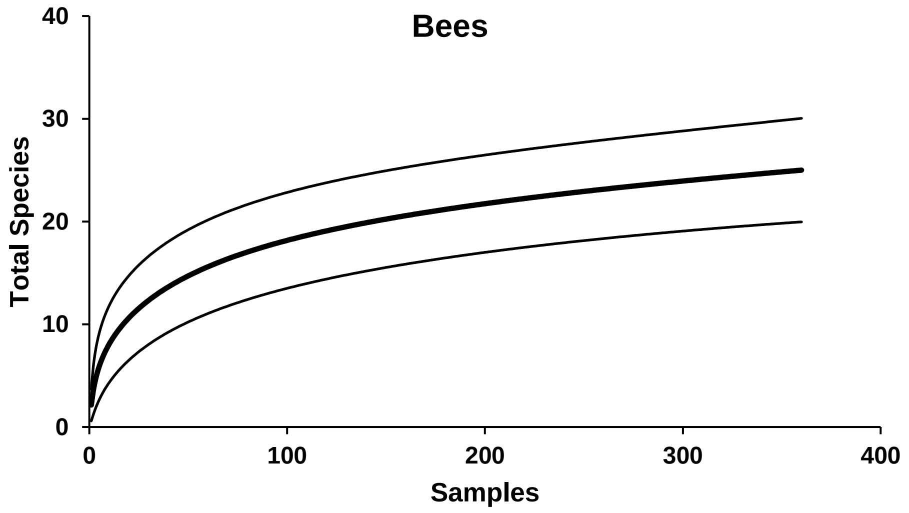
Bees
40
30
20
10
0
Total Species
0
100
200
300
400
Samples

B)

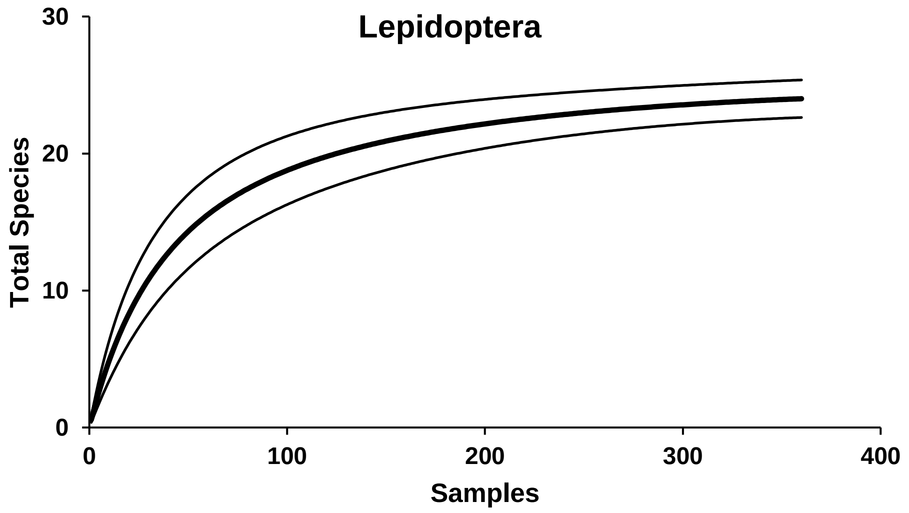
Lepidoptera
30
20
10
0
Total Species
0
100
200
300
400
Samples

C)

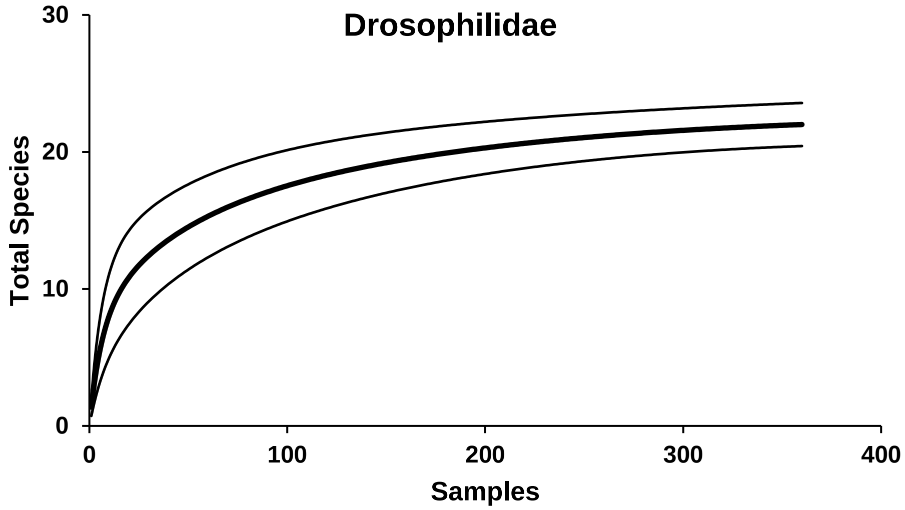

Drosophilidae
Total Species
Samples
30
20
10
0
0
100
200
300
400

D)

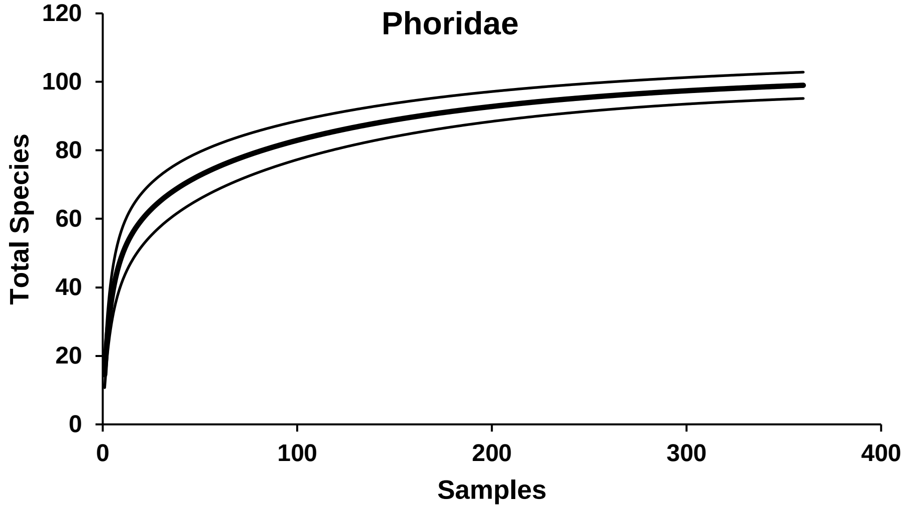

Phoridae
Total Species
Samples
120
100
80
60
40
20
0
0
100
200
300
400

E)

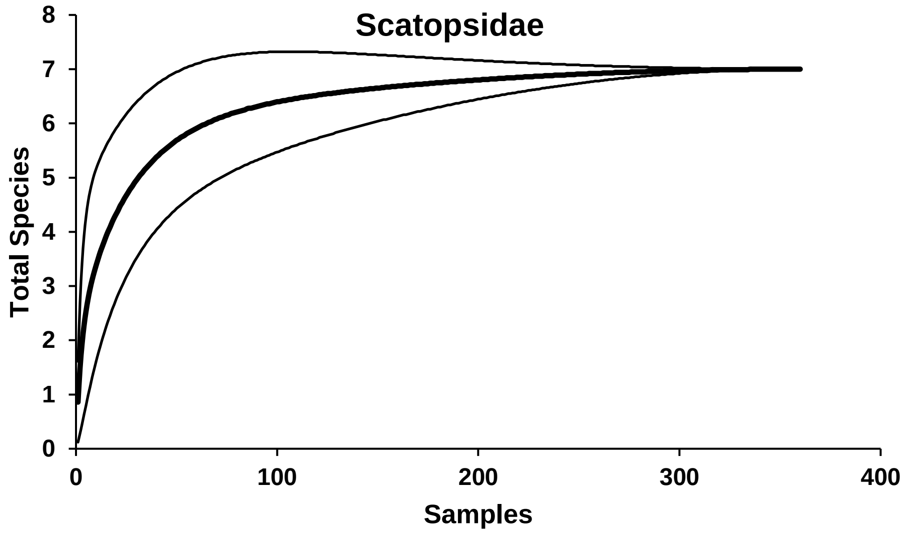
Scatopsidae
Total Species
8
7
6
5
4
3
2
1
0
0
100
200
300
400
Samples

F)

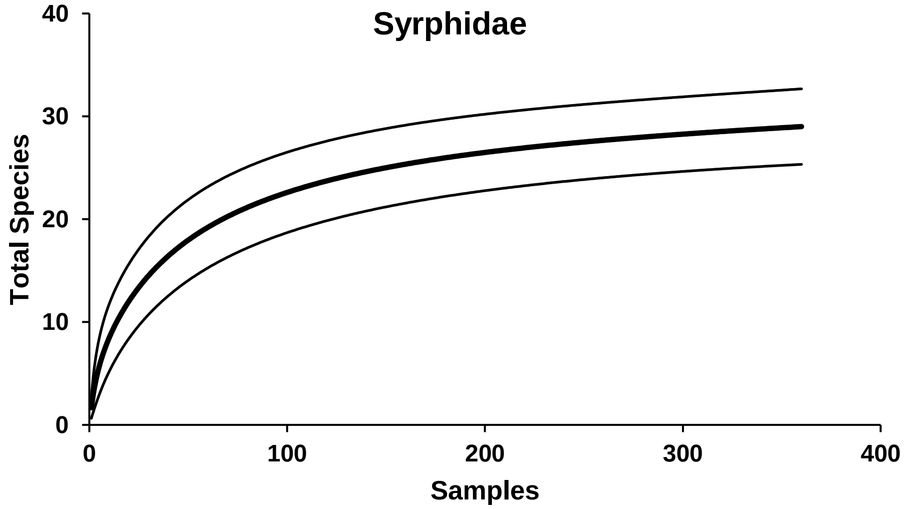
Syrphidae
Total Species
40
30
20
10
0
0
100
200
300
400
Samples

G)

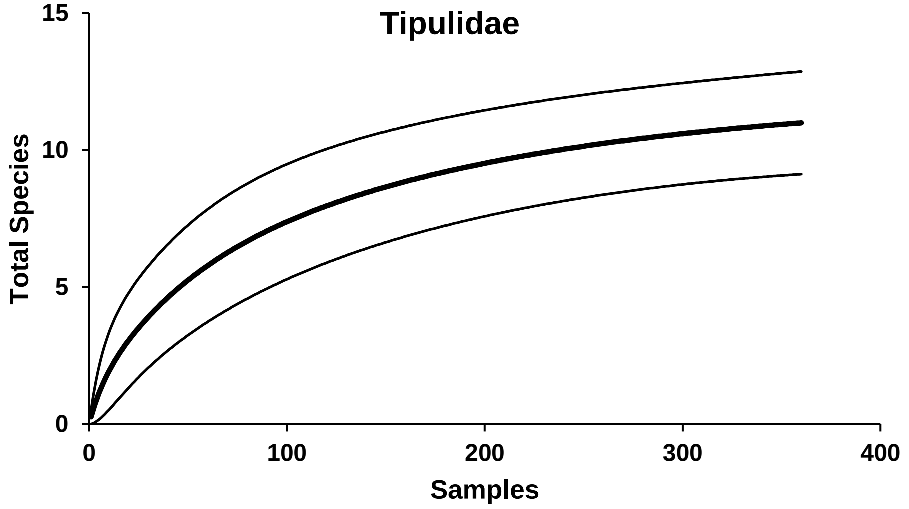

Figure S4.

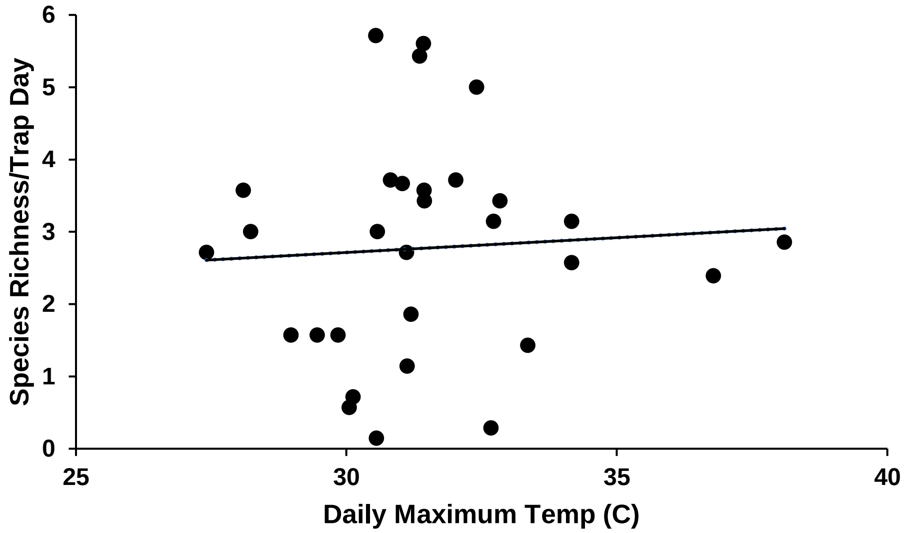

Species Richness/Trap Day
6
5
4
3
2
1
0
25
30
35
40
Daily Maximum Temp (C)

Figure S5.

A)

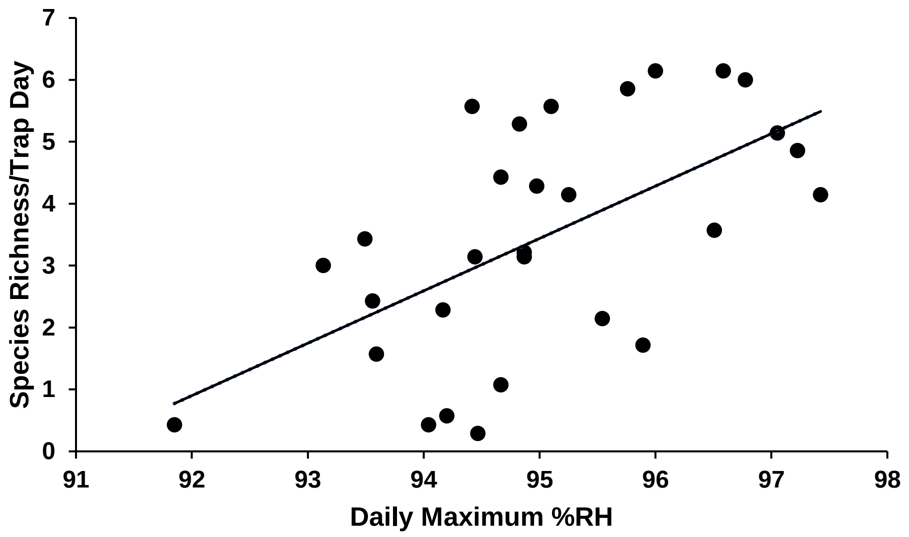

Species Richness/Trap Day
Daily Maximum %RH

B)

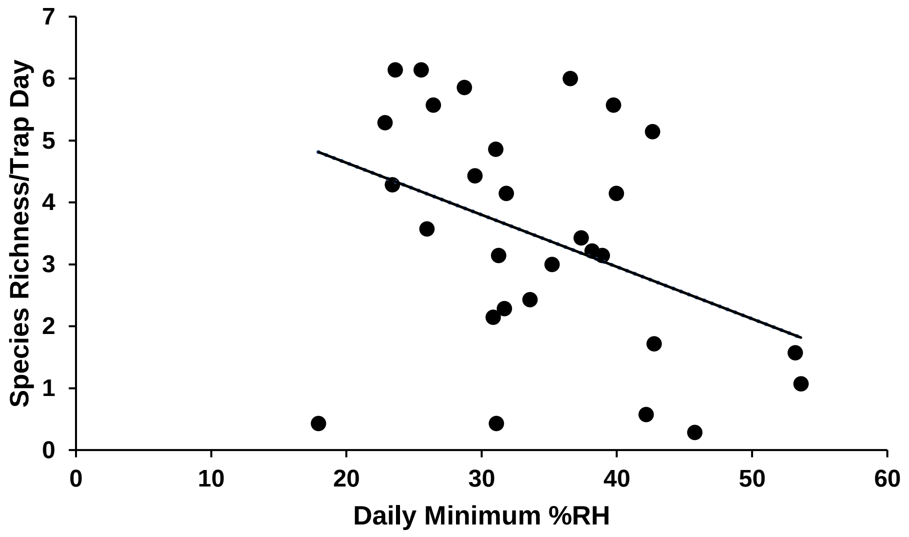

Species Richness/Trap Day
Daily Minimum %RH

Figure S6.

A)

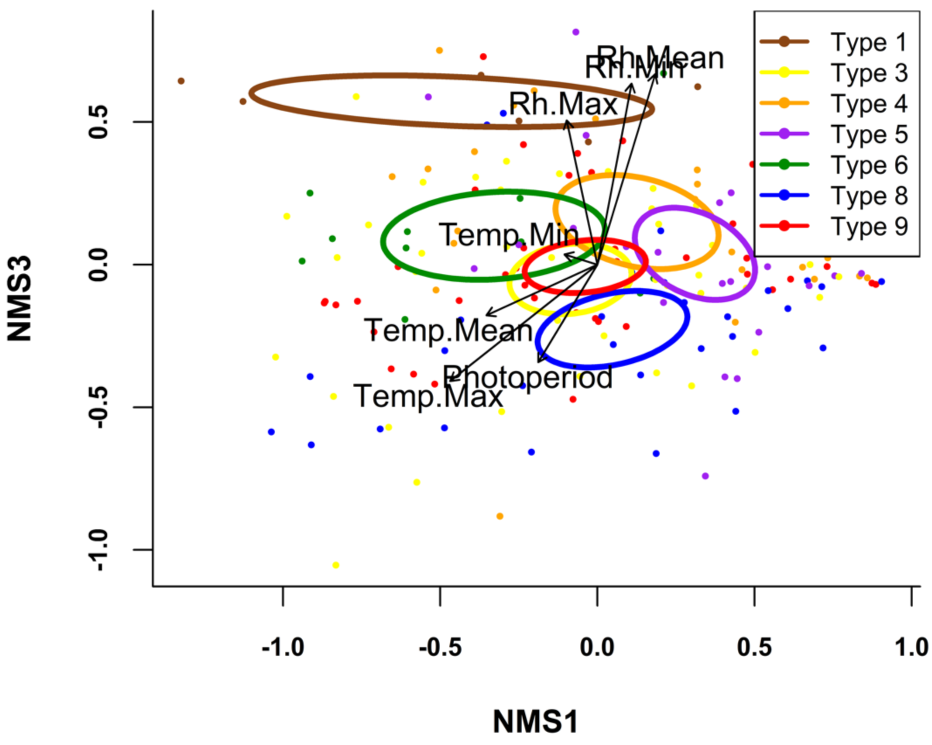
Type 1
Type 3
Type 4
Type 5
Type 6
Type 8
Type 9
Rh.Mean
Rh.Min
Rh.Max
Temp.Min
Temp.Mean
Photoperiod
Temp.Max
NMS3
NMS1
0.5
0.0
-0.5
-1.0
-1.0
-0.5
0.0
0.5
1.0

B)

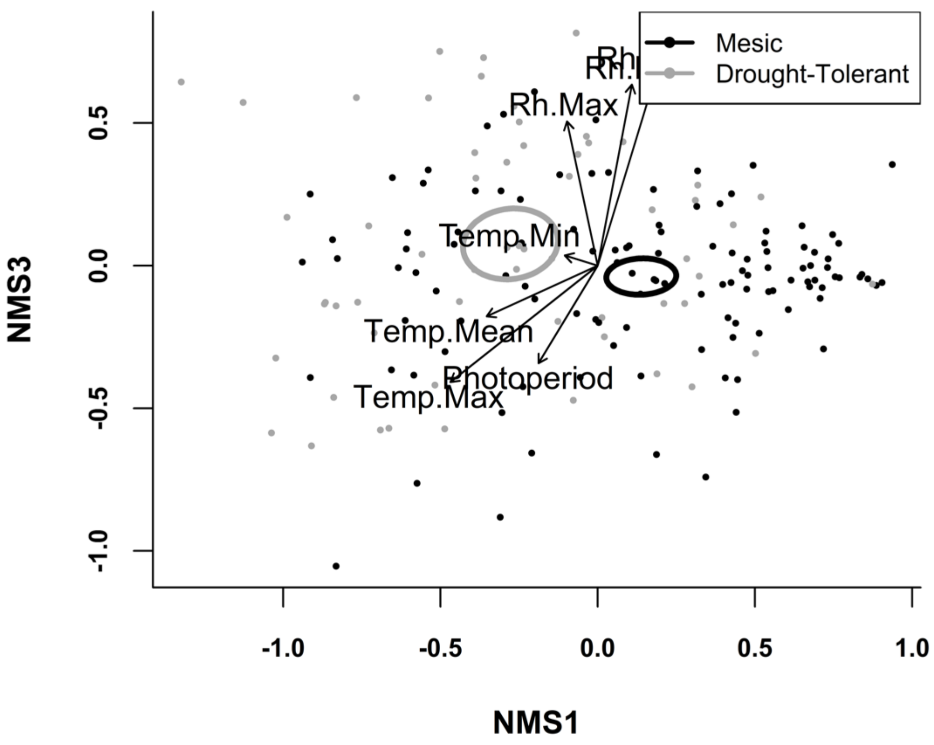
Mesic
Drought-Tolerant
Rh.Max
Temp.Min
Temp.Mean
Photoperiod
Temp.Max
NMS3
NMS1
0.5
0.0
-0.5
-1.0
1.0

C)

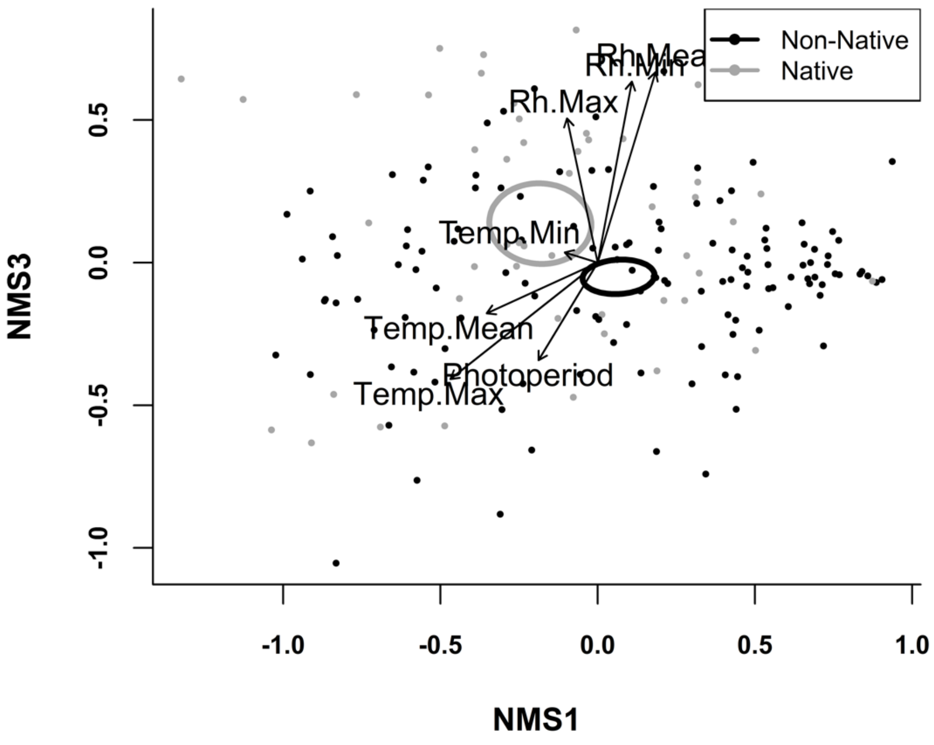
Non-Native
Native
Rh.Min
Rh.Max
Temp.Min
Temp.Mean
Photoperiod
Temp.Max
NMS3
NMS1
0.5
0.0
-0.5
-1.0
1.0

D)

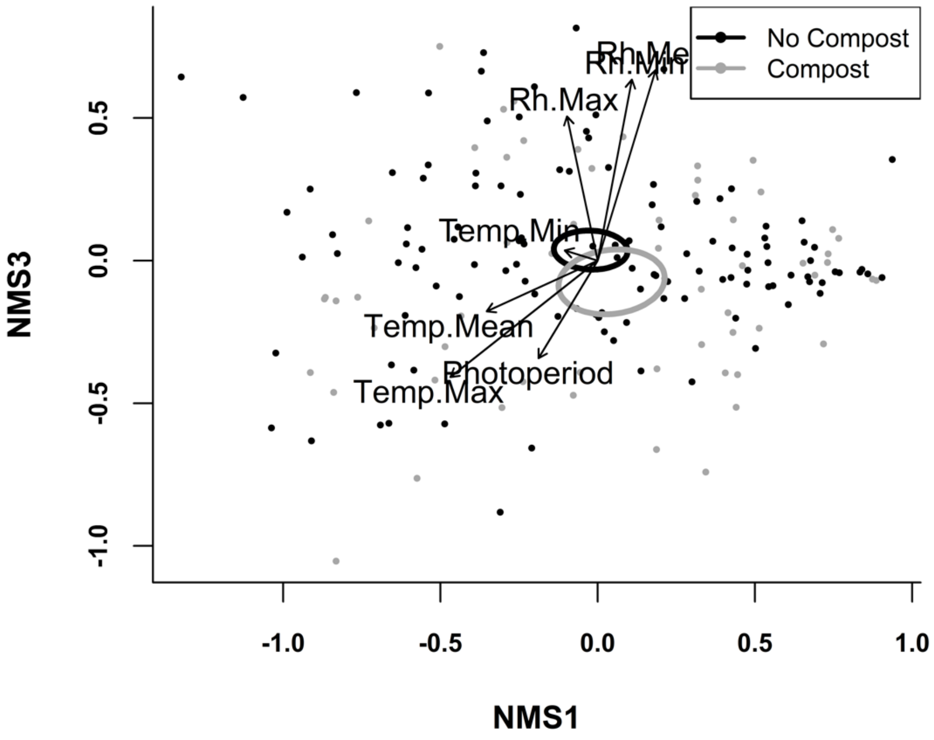

No Compost
Compost
Rh.Max
Temp.Min
Temp.Mean
Photoperiod
Temp.Max
0.5
0.0
-0.5
-1.0
NMS3
-1.0
-0.5
0.0
0.5
1.0
NMS1

Figure S7.

A)

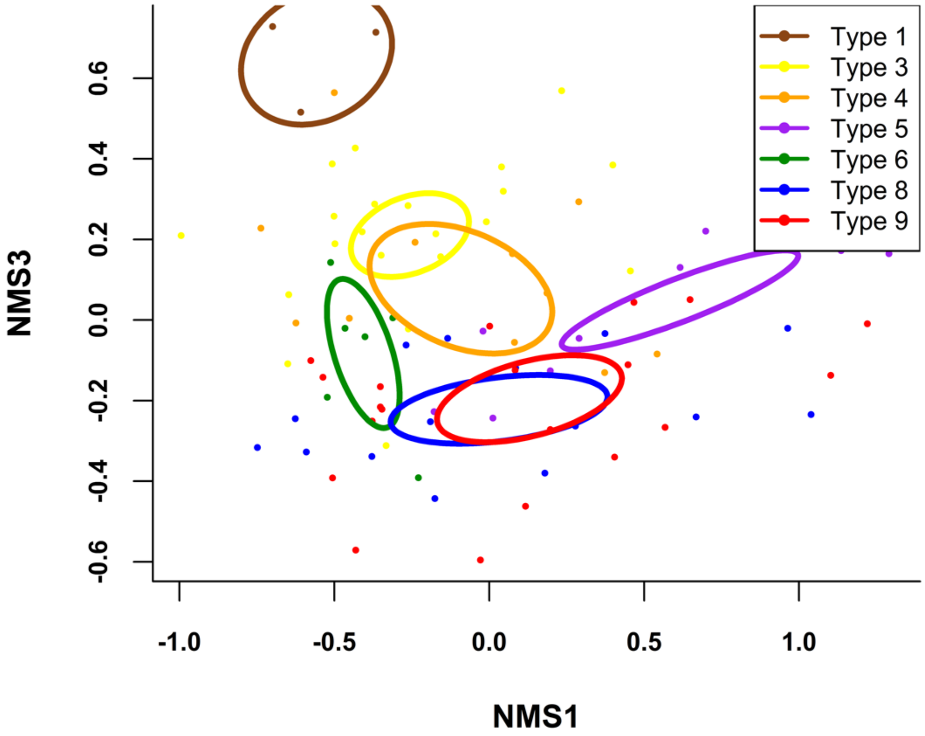

Type 1
Type 3
Type 4
Type 5
Type 6
Type 8
Type 9
NMS3
NMS1
0.6
0.4
0.2
0.0
-0.2
-0.4
-0.6
-1.0
-0.5
0.0
0.5
1.0

B)

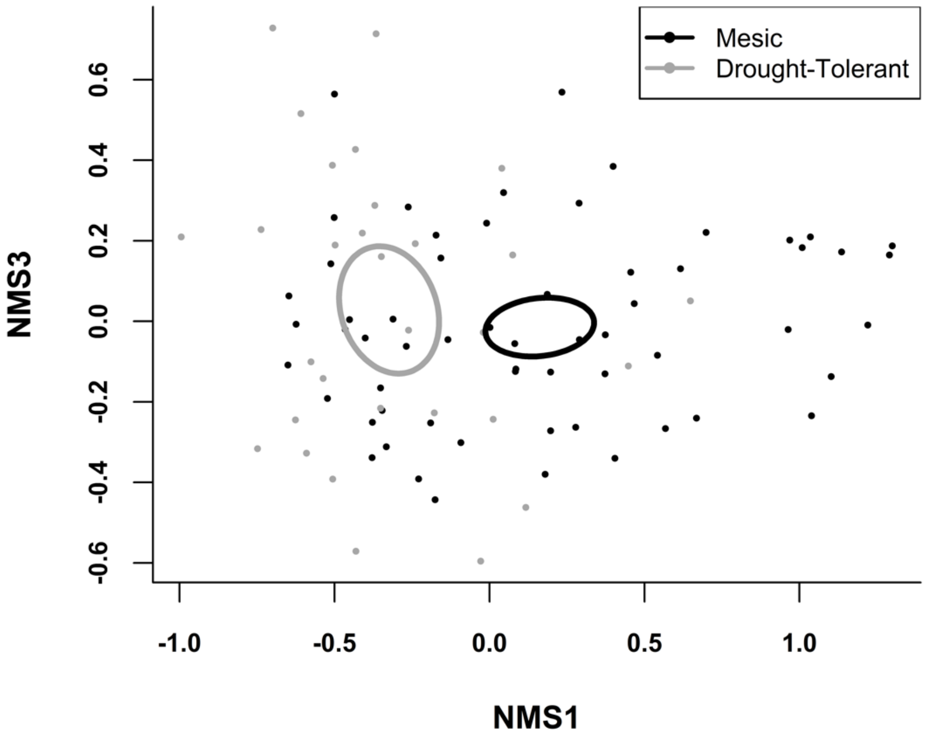

Mesic
Drought-Tolerant
NMS3
NMS1
0.6
0.4
0.2
0.0
-0.2
-0.4
-0.6
-1.0
-0.5
0.0
0.5
1.0

C)

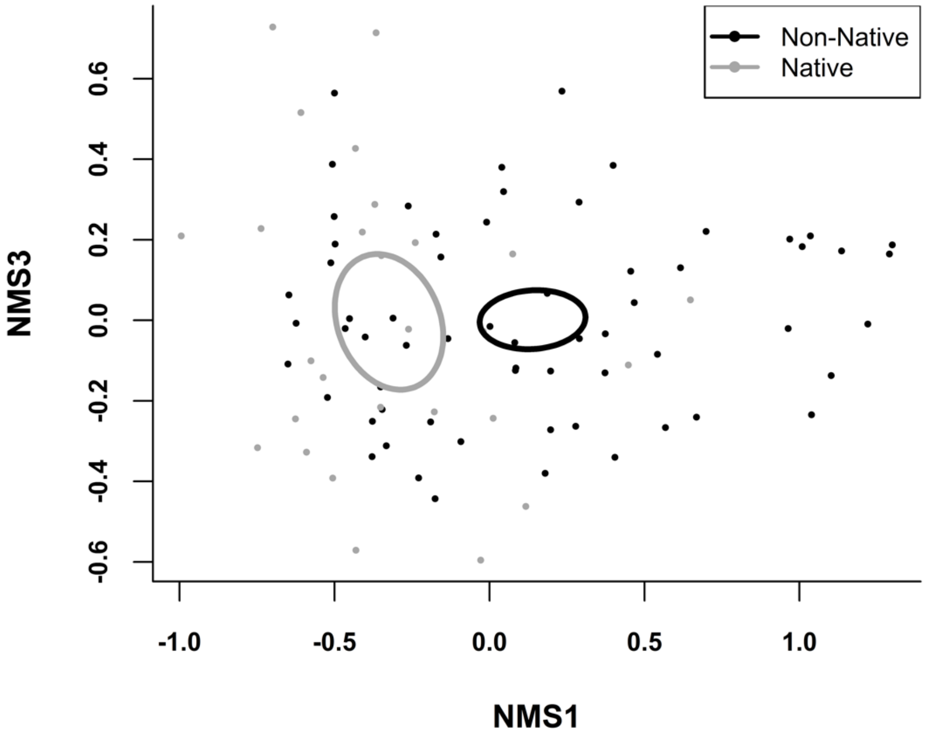

Non-Native
Native
NMS3
NMS1
0.6
0.4
0.2
0.0
-0.2
-0.4
-0.6
-1.0
-0.5
0.0
0.5
1.0

Figure S8.

A)

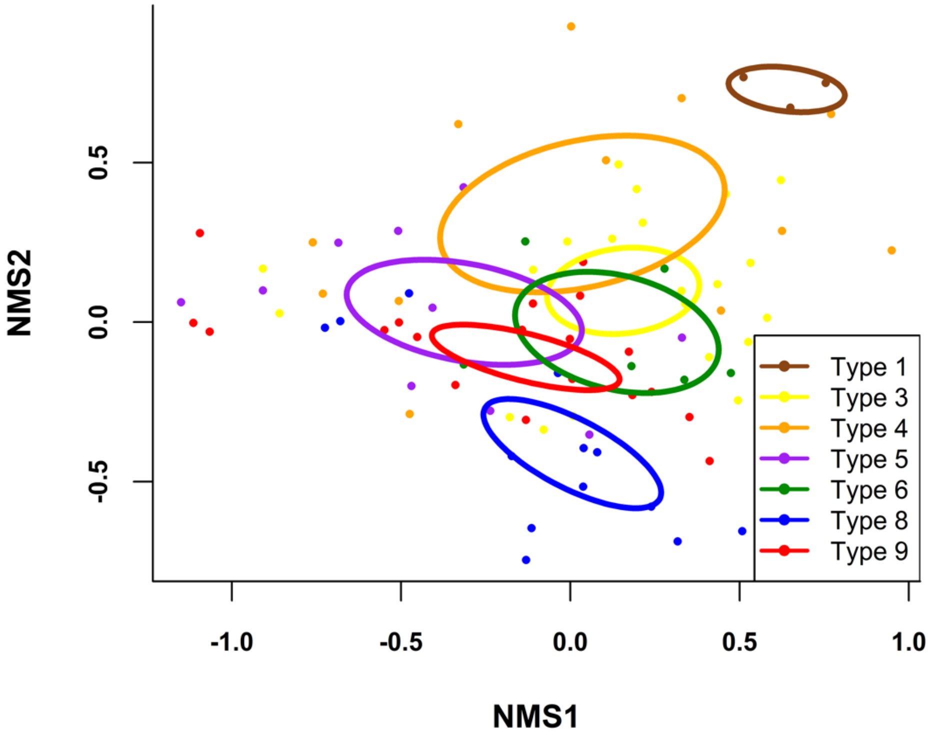

NMS2
NMS1
0.5
0.0
-0.5
-1.0
-0.5
0.0
0.5
1.0
Type 1
Type 3
Type 4
Type 5
Type 6
Type 8
Type 9

B)

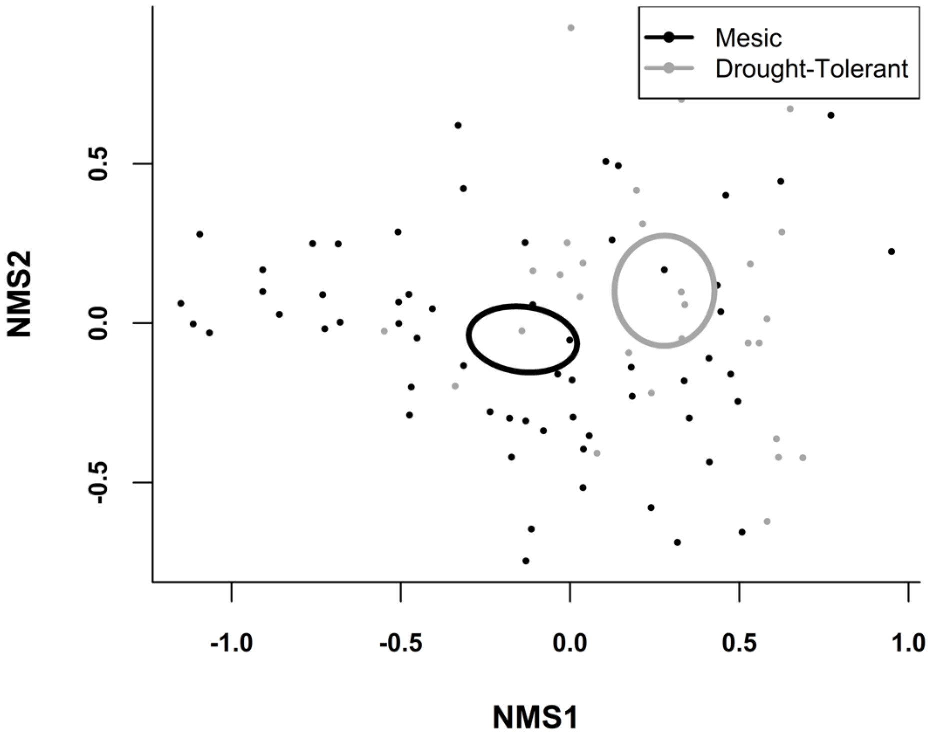
Mesic
Drought-Tolerant
NMS2
0.5
0.0
-0.5
-1.0
-0.5
0.0
0.5
1.0
NMS1

C)

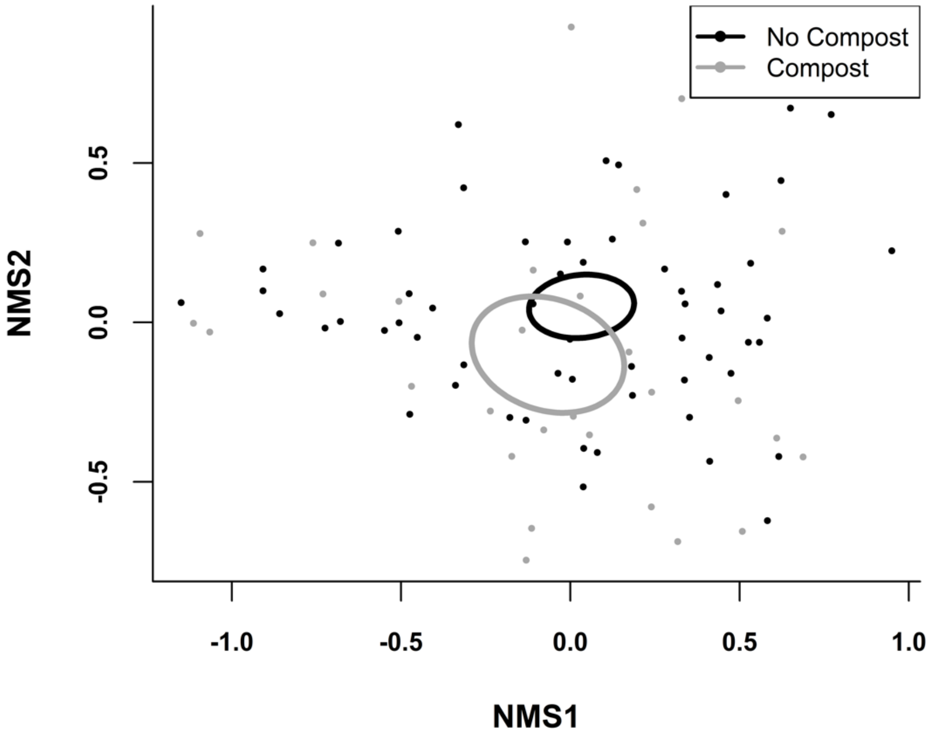
No Compost
Compost
NMS2
0.5
0.0
-0.5
-1.0
-0.5
0.0
0.5
1.0
NMS1

Literature Citations

de Cáceres, M. D., and P. Legendre. 2009. Associations between species and groups of sites: indices and statistical inference. Ecology 90:3566-3574.

Dufrêne, M., and P. Legendre. 1997. Species assemblages and indicator species: the need for a flexible asymmetrical approach. Ecological Monographs 67:345-366.
